# Downregulation of miRNA miR-1305 and upregulation of miRNA miR-6785-5p may be associated with psoriasis

**DOI:** 10.3389/fgene.2022.891465

**Published:** 2022-08-10

**Authors:** Jianjun Yan, Yunyue Zhen, Ruijie Wang, Xueqing Li, Shan Huang, Hua Zhong, He Wen, Qing Sun

**Affiliations:** ^1^ Department of Dermatology, Qilu Hospital of Shandong University, Jinan, China; ^2^ Laboratory of Basic Medical Science, Qilu Hospital of Shandong University, Jinan, China

**Keywords:** extracellular vesicles, miR-1305, MiR-6785-5p, psoriasis, inflammation

## Abstract

**Background:** The role of serum extracellular vesicles (EVs) is less known in psoriasis.

**Objectives:** To explore the transcriptomic profile of serum EVs and the potential biomarkers in psoriasis.

**Methods:** EVs were isolated by differential ultracentrifugation and identified by transmission electron microscope. The diameters of EVs were detected using nanoparticle tracking analysis. Serum EVs-keratinocyte interaction was observed through confocal fluorescence microscopy. miRNA microarray and mRNA microarray were performed in serum EVs (*n* = 4) and skin lesions (*n* = 3), respectively. Quantitative reverse-transcriptase polymerase chain reaction (qRT-PCR) and fluorescence *in situ* hybridization were used to detect the expression of miRNAs in serum EVs and skin lesions (*n* = 15). Bioinformatics analysis was performed to predict the potential target genes and functions of miR-1305 and miR-6785-5p. Western blot, CCK-8 and enzyme-linked immunosorbent assay (ELISA) were used to detect the EVs’ biomarkers, keratinocytes proliferation and cytokines secretion.

**Results:** A total of 16 miRNAs and 1,725 mRNAs were significantly dysregulated in serum EVs and skin lesions, respectively. miR-1305 was down-regulated and miR-6785-5p was upregulated in both serum EVs and skin lesions. Serum EVs could be taken up by keratinocytes. miR-1305 was downregulated and miR-6785-5p were upregulated in keratinocytes after co-cultured with psoriasis serum EVs compared with controls. Psoriasis serum EVs promoted keratinocyte proliferation and the secretion of CCL20 and IL-8. Serum EVs miR-1305 and miR-6785-5p were associated with disease severity.

**Conclusion:** Serum EVs might be involved in the activation of keratinocytes through loaded miRNAs in psoriasis. Serum EVs miR-1305 and miR-6785-5p may be associated with psoriasis.

## Introduction

Psoriasis is a chronic inflammatory skin disease (Srivastava et al., 2017; Nussbaum et al., 2021). The worldwide prevalence rate of this disease is about 2%–3% (Boehncke and Schon, 2015). Multiple factors contribute to the skin inflammation of psoriasis, including genetic, environmental, and immunologic triggers (Frischknecht et al., 2019; Griffiths et al., 2021). Psoriasis was proposed to result from a complex interplay among keratinocytes, immune cells and inflammatory mediators (Guinea-Viniegra et al., 2014). Keratinocytes are the main constituents of the epidermis and play an important role in the formation of psoriatic skin inflammation (Ni and Lai, 2020; Chen et al., 2021). Previous studies have suggested that the intercellular communication between keratinocytes and immune cells is mediated by proinflammatory cytokines (Than et al., 2019; Papayannakos et al., 2021). CCL20 and IL-8 secreted by keratinocytes play important roles in psoriasis, including the activation and chemotaxis of Th17 cells and neutrophils (Tsai and Tsai, 2017; Furue et al., 2020).

Recently, extracellular vesicles (EVs) are emerging mediators of intercellular communication between keratinocytes and immune cells, thought to participate in the pathogenesis of psoriasis (Shao et al., 2020). EVs are secreted from numerous cell types and have been isolated from a wide variety of human body fluids such as blood, urine, and saliva (Kim et al., 2017). EVs represent an important mode of intercellular communication by serving as vehicles for transfer between cells of membrane and cytosolic proteins, lipids, and RNA. Its functions depend on the ability of EVs to interact with recipient cells to deliver their contents (Raposo and Stoorvogel, 2013). A previous study has shown that keratinocyte-derived EVs promoted the Th1/Th17 polarization in psoriasis (Jiang et al., 2021). However, the characterization, transcriptomic profile and functional role of serum EVs in mediating keratinocyte activation and psoriatic skin inflammation are yet to be explored.

MicroRNAs (miRNAs) are 18–22-nucleotide, single-stranded, endogenous noncoding small RNA molecules. MiRNAs target mRNAs through complementary base pairing with 3′-UTRs of the target transcripts, in either a complete or incomplete fashion (Lu and Rothenberg, 2018). MiRNAs function by inhibiting mRNA translation and stability. Recently, miRNA dysregulation in the pathogenesis of psoriasis has attracted attention (Wu et al., 2018). However, little is known about the transcriptomic profile of miRNAs in serum EVs and the role of serum EVs miRNAs in the activation of keratinocytes in psoriasis.

Previous studies have confirmed the intrinsic cell targeting properties of EVs (Vader et al., 2016). However, recent studies mainly focused on the communication between EVs and neighbouring cells in psoriasis, such as T cells or neutrophils (Cheung et al., 2016; Shao et al., 2019). The communication between serum EVs and keratinocytes remains less known. As the cell-targeting properties of EVs and the serum EVs-keratinocyte interaction were confirmed in this study, we assume that serum EVs might not only communicate with neighbouring cells but also communicate with keratinocytes in psoriasis.

In this study, we detected the characterization of serum EVs from psoriasis patients and healthy controls, and verified serum EVs could be taken up by keratinocytes through confocal fluorescence microscopy (CFM). To investigate the regulatory role of serum EVs in the activation of keratinocytes, we isolated serum EVs and performed a miRNA microarray. The mRNA microarray was also performed in skin lesions. In addition, we verified the role of serum EVs and the expression of serum EVs miR-1305 and miR-6785-5p. Previous studies suggest that miR-1305 and miR-6785-5p play important roles in diseases by regulating TGF-β2/smad3 pathway, Wnt/beta-catenin pathway, Akt-signaling pathway and so on (Yu et al., 2019; Liu et al., 2020; Su et al., 2020; Kim et al., 2021). Furthermore, we found miR-1305 and miR-6785-5p might be involved in regulating inflammation response and correlated with the disease severity of psoriasis.

Our findings indicated a potential role of serum EVs in the epidermal hyperplasia and skin inflammation in psoriasis, opening the possibility that the serum EVs miRNAs might be as biomarkers and that serum EVs-keratinocytes interaction can be harnessed as a platform for innovative therapeutic strategies in psoriasis.

## Materials and methods

### Patients and tissue samples

Fifteen specimens were obtained from patients with psoriasis vulgaris (ten males and five females, aged 25–59 years). They had received no systemic treatments, phototherapy or externally used drugs for at least 1 month before skin biopsies and blood specimens collected. Ten control skin biopsies were obtained from healthy volunteers (five males and five females, aged 22–57 years). We obtained biopsies (1 × 0.5 cm) by surgical operations and whole blood (5–10 ml). Psoriasis Area Severity Index (PASI) was determined at the condition when samples were collected by at least two dermatologists. The study was approved by the ethics committee of Shandong University, China, and all patients provided written informed consent.

### Serum extracellular vesicles extraction

Whole blood (5–10 ml) from patients and healthy controls were centrifuged at 3,000 rpm for 10 min to separate serum. The serum was collected, centrifuged at 300 g for 10 min, then at 16,500 g for 30 min, and later filtered through a 0.22 mm pore size filter to remove cell debris. The final supernatant was ultracentrifuged at 100,000 g for 70 min and recentrifuged at the same speed. The purified EVs were resuspended in PBS.

### Characterization of serum extracellular vesicles

Morphology of serum EVs was observed by transmission electron microscopy (TEM). The numbers and diameters were detected using nanoparticle tracking analysis (NTA, Zeta-View PMX 110; Particle Metrix, Meerbusch, Germany). Phenotypic was assessed by western blot using antibodies (Abcam) specific for CD9 (1:2,000, ab92726), CD63 (1:1,000, ab134045), and CD81 (1:1,000, ab109201).

### miRNA and mRNA microarray analysis

Serum EVs miRNAs and mRNAs in skin lesions were detected using Agilent Human miRNA microarray V 21.0 (Sinotech Genomics, Shenzhen, China) and SBC Human (4 × 180K) competing endogenous (ce)RNA microarray (Shanghai Biotechnology Corporation, Shanghai, China), respectively. The fold change >2 in these analyses was an absolute value of fold change.

### Fluorescence *in situ* hybridization analysis

A diluted double CY3-labelled miR-1305 probe (8 ng/μl, 5′-CY3-AAAAGTTGAGATTACCCTCTCT-CY3-3’; Servicebio, Wuhan, China) and miR-6785-5p probe (8 ng/μl, 5′-CY3- ACCCTCCCGCACCTACTACCAC-CY3-3’; Servicebio, Wuhan, China) were used for hybridization. 4′,6-Diamidino-2-phenylindole (Servicebio, Wuhan, China) was used for nuclear staining.

### Serum extracellular vesicles-keratinocytes interaction experiment

Serum EVs were labelled with PKH26 Red Fluorescent Cell Linker Mini kit (MINI26-1 KT; Sigma-Aldrich, St. Louis, MO) according to the instructions. Then, the suspension was centrifuged at 100,000 g for 70 min, and the supernatant was discarded. The labelled EVs were resuspended in PBS and centrifuged once again at the same speed. Next, the EVs were co-cultured with normal human epidermal keratinocytes (NHEKs) cells for 12 h. Then washed the cells 3 times with PBS and fixed with 4% formaldehyde. Subsequently, the cells were stained using phalloidin-iFluor 488 reagent, and DAPI was added to stain the nuclei. The cells were then observed under a confocal microscope (LSM800; Zeiss).

### Quantitative reverse-transcriptase polymerase chain reaction

Serum EVs were digested with RNaseA (100 ng/ml, Qiagen, Australia). Then, total RNA from serum EVs was extracted using TRIzol reagent (Invitrogen). The expression levels of serum EVs miRNAs and miRNAs in skin lesions were detected according to the manual of the All-in-One miRNA qRT-PCR Detection System (GeneCopoeia, Guangzhou, China). U6 was used as the internal control for miRNA. Total RNAs from skin samples were extracted using an RNeasy mini kit (cat. no. 74106; Qiagen GmBH, Hilden, Germany) according to the manufacturer’s protocols. Then the expression levels of mRNAs were detected using microarray analysis.

### Western blot analysis

Western blot was performed as described in our previous experiments (Jiang et al., 2017; Zhao et al., 2017). The following primary antibodies were used in this study: rabbit monoclonal antibodies (procured from Abcam) specific for CD9 (1:2,000, ab92726), CD63 (1:1,000, ab134045) and CD81 (1:1,000, ab109201) and GAPDH (1:1,000, ab8245).

### Bioinformatics analysis and miRNA-mRNA net-work analysis

Target genes of miRNAs were predicted using TargetScanHuman 7.1 and miRDB-MicroRNA Target Prediction Database. Then, the genes were intersected with mRNAs detected by mRNA microarray. Enrichment calculations were performed using Fisher’s exact test. Further, we conducted GO and pathway enrichment analysis of the target genes. The specific principle was to carry out annotation mapping of differentially expressed genes in GO and KEGG database entries, calculate the number of the target genes in each GO and pathway entry, and then use hypergeometric test for statistics. Select the GO and KEGG entries that were significantly enriched in the differentially expressed genes. After the calculated *p*-value was corrected by multiple hypothesis tests, the *p*-value 0.05 was taken as the threshold, and the GO and KEGG term meeting this condition was defined as the GO and KEGG term significantly enriched in the target genes. MiRNA-mRNA network analysis was visualized using Cytoscape 3.9.0 software.

### CCK-8 proliferation assay

The proliferation rates of NHEK Cells were measured at different time intervals using CCK-8 assays (Beyotime, Shanghai, China) after co-cultured with serum EVs obtained from psoriasis patients (*n* = 15) and healthy controls (*n* = 10) for 12 h. Optical density (OD) values at 450 nm were measured using a Synergy H1 Microplate Reader (BioTek, United States).

### Enzyme-linked immunosorbent assay assay

After 24 h co-cultured with serum EVs, the culture supernatants were collected. Then, the expression levels of CCL20 and IL-8 secreted from NHEK cells were measured using specific ELISA kits (Elabscience, China). Absorbance at 450 nm was measured using an ELISA plate reader (Bio-Rad).

### Statistical analysis

All data are presented as mean ± standard deviation. All experiments were performed at least 3 times. The differences between 2 groups were analyzed using Student’s t-test with SPSS 17_0 analytical software (IBM, Armonk, NY, United States) and GraphPad Prism 7.00 (GraphPad Software, La Jolla, CA, United States). The target genes in GO and pathway were using hypergeometric test for statistics and *p*-value was corrected by multiple hypothesis tests. *p*-values < 0.05 were considered significant.

## Results

### Isolation and characterization of serum extracellular vesicles

**FIGURE 1:**
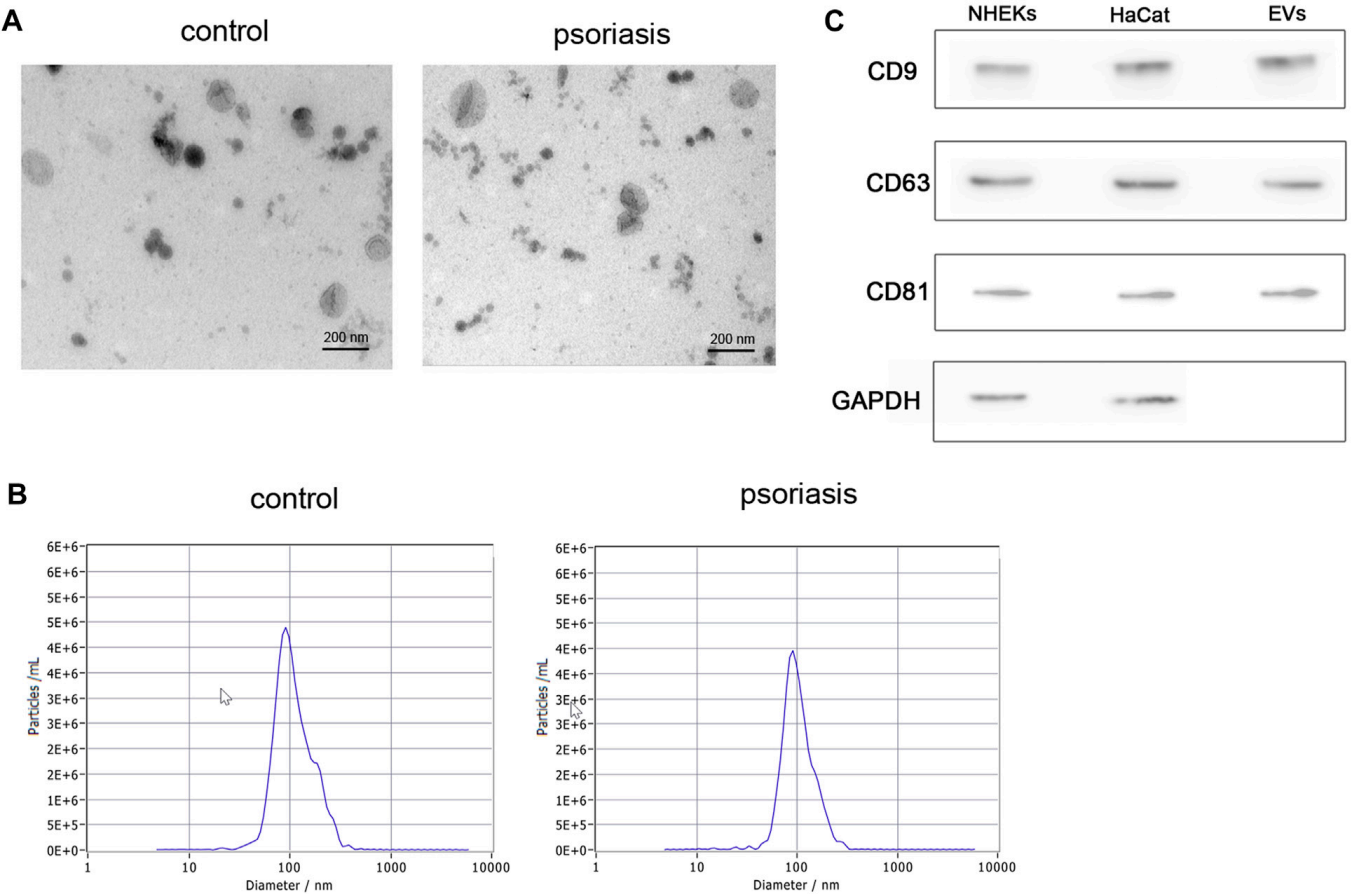
Isolation and characterization of serum EVs. **(A)** Serum EVs were observed using transmission electron microscopy (scale bar, 200 nm). Serum EVs exhibited a typical artificial cup shape appearance; **(B)** the diameter of serum EVs was detected by nanoparticle tracking analysis (*n* = 3); **(C)** surface marker proteins of serum EVs were analyzed by western blot (*n* = 3), NHEKs and HaCat cells were used as poistive controls. EVs: extracellular vesicles, scale bar = 200 nm.

To investigate the characterization of serum EVs in psoriasis, we isolated serum EVs from psoriasis patients and healthy controls using differential ultracentrifugation. Using the methods of transmission electron microscopy (TEM) and nanoparticle tracking analysis (NTA), we confirmed that purified serum EVs obtained from psoriasis patients and healthy controls both exhibited a typical cup-shaped structure (Figure 1A), and their average diameters were around 100 nm (Figure 1B). In addition, as shown in Figure 1C, the expression of CD9, CD63, and CD81 was identified in serum EVs by western blot analysis.

### Serum extracellular vesicles from psoriasis patients exhibit a specific miRNA profile

**FIGURE 2:**
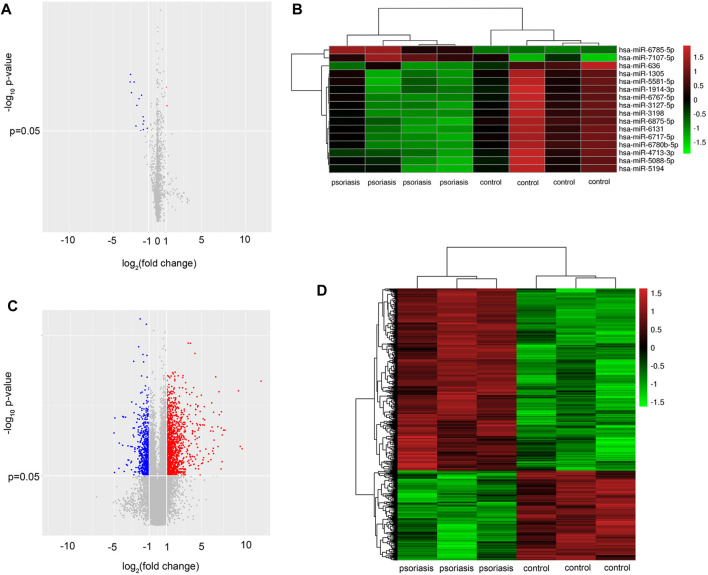
Serum EVs from psoriasis patients exhibit a specific miRNA profile. **(A)** Volcano plot of differentially expressed miRNAs from serum EVs between psoriasis and normal control (*n* = 4); **(B)** Hierarchical clustering analysis of the differentially expressed miRNAs (*n* = 4). Red and green colours indicated high and low expression, respectively; **(C)** volcano plot of differentially expressed mRNAs in skin lesions between psoriasis and normal control (*n* = 3). **(D)** Hierarchical clustering analysis of the differentially expressed mRNAs (*n* = 3). Red and green colours indicated high and low expression, respectively. miRNA: microRNA; EVs: extracellular vesicles.

To determine miRNA profiling of serum EVs in psoriasis, the expression of EVs miRNAs was detected using miRNA microarray analysis (*n* = 4). The results showed remarkable differences in the serum EVs miRNA profiles, with 16 differentially expressed miRNAs in serum EVs from psoriasis compared with healthy controls (GSE200637). Among 16 differentially expressed miRNAs, 2 were upregulated and 14 were down-regulated (fold change>2, *p* < 0.05, Figures 2A,B), (Supplementary Table S1).

To further investigate the potential role of serum EVs miRNAs in the activation of keratinocytes, we performed mRNA microarray analysis in psoriatic skin lesions and healthy controls (*n* = 3). According to the mRNA microarray results, a total of 1,725 dysregulated mRNAs were identified. Among 1,725 dysregulated mRNAs, 1,157 mRNAs were upregulated and 568 mRNAs were downregulated (fold change>2, *p* < 0.05, Figures 2C,D), (Supplementary Table S2). The mRNA microarray results suggested that there were a greater number of upregulated mRNAs in psoriatic skin lesions compared with healthy controls, which corresponded to the majority of miRNAs that were downregulated in serum EVs.

### miRNA microarray validation

**FIGURE 3:**
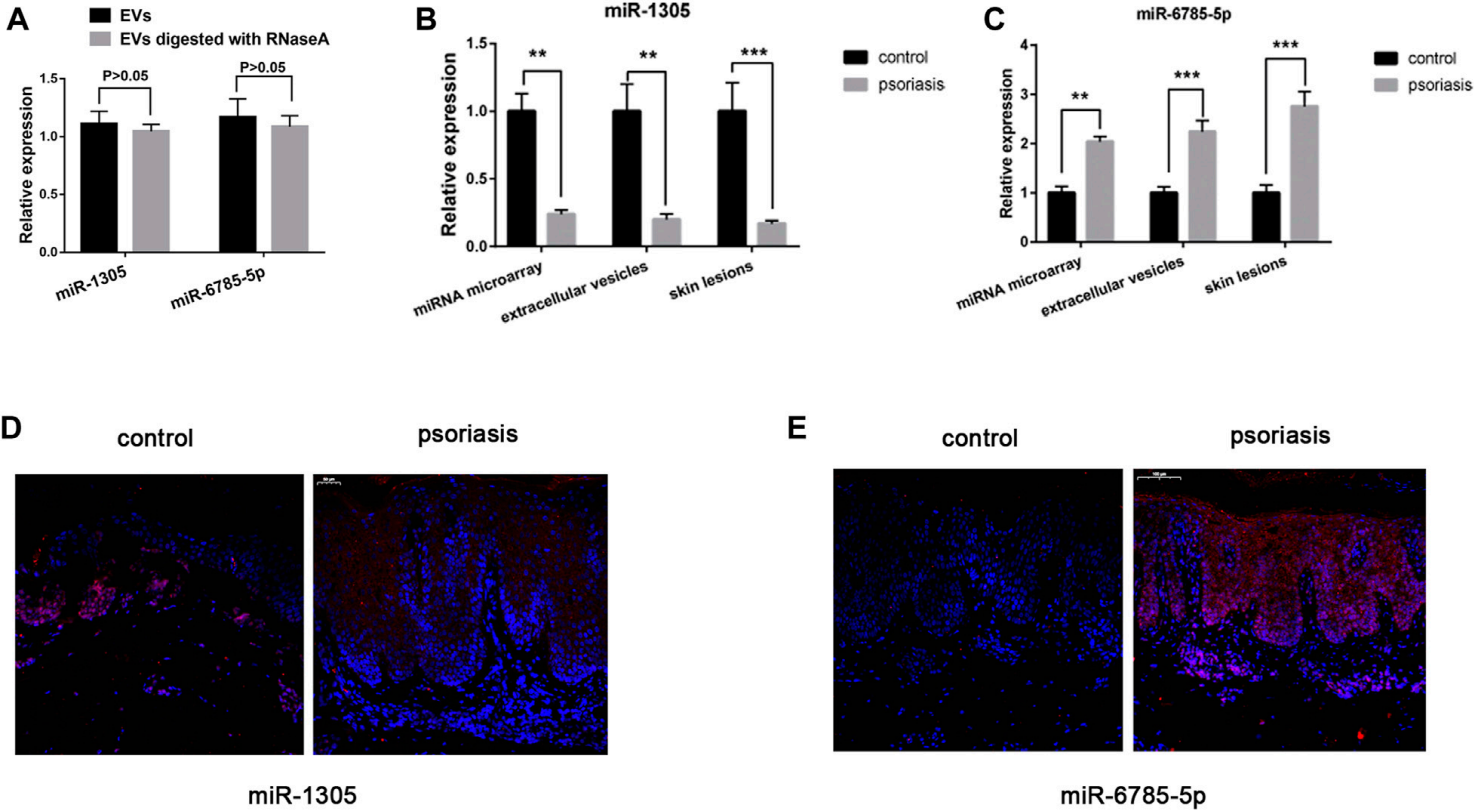
MiRNA microarray validation. **(A)** EVs digested with or without RNaseA, and miR-1305 and miR-6785-5p were detected by qRT-PCR. **(B)**The expression of miR-1305 was detected by miRNA microarray and validated in serum EVs and skin lesions respectively (*n* = 10). **(C)** The expression of miR-6785-5p was detected by miRNA microarray, and validated in serum EVs and skin lesions respectively (*n* = 10). **(D,E)** The expression of miR-1305 and miR-6785-5p in skin lesions were detected by FISH (*n* = 10). miRNA: microRNA; scale bar = 100 µm. EVs: extracellular vesicles; FISH: Fluorescence *in situ* hybridization analysis. **indicates *p* < 0.01 and ***indicates *p* < 0.001.

To validate the miRNA microarray results, we performed qRT-PCR (*n* = 10) and Fluorescence *in situ* hybridization (*n* = 10) to detect the expression of miR-1305 and miR-6785-5p which predicted closely related with inflammation responses. We digested EVs with RNaseA and reperformed qRT-PCR, the results indicated that miR-1305 and miR-6785-5p are present inside of EVs not on the surface of EVs (Figure 3A). The results of Figures 3B,C showed that the expression of miR-1305 was down-regulated in both serum EVs and skin lesions (fold change = 0.2 in EVs, *p* < 0.01; fold change = 0.17 in skin lesions, *p* < 0.001) and miR-6785-5p was upregulated (fold change = 2.24 in EVs, *p* < 0.001; fold change = 2.76 in skin lesions, *p* < 0.001). As shown in Figures 3D,E, the results of fluorescence *in situ* hybridization (FISH) were consistent with qRT-PCR and showed that miR-1305 and miR-6785-5p were differentially expressed in the epidermis.

### Potential functions of miR-1305 and miR-6785-5p

**FIGURE 4:**
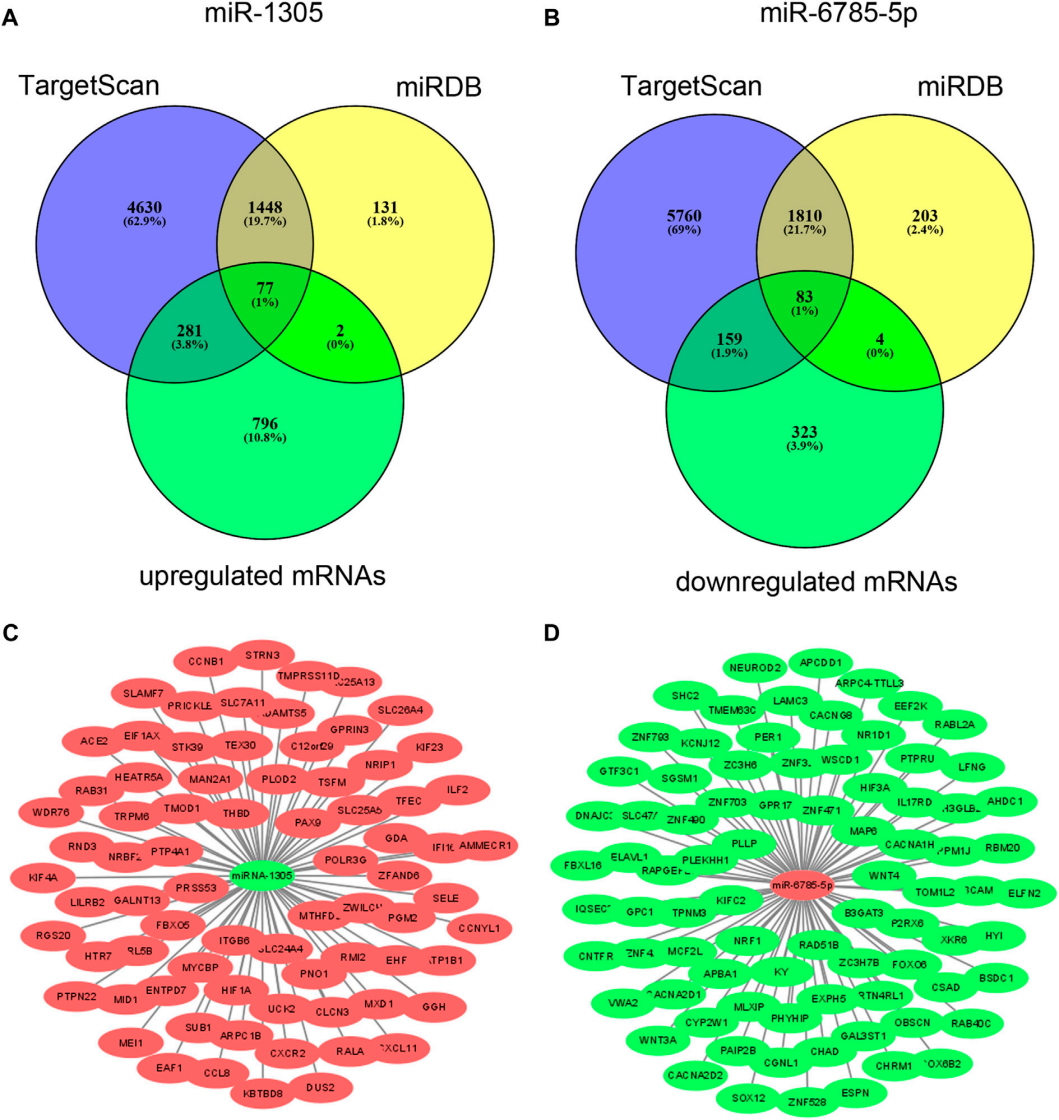
Predicted target genes of miR-1305 and miR-6785-5p, and miRNA-mRNA network analysis. **(A,B)** Target genes of miR-1305 and miR-6785-5p were predicted by TargetScan and miRDB, and then intersected with upregulated and downregulated mRNAs which detected by mRNA microarray respectively. **(C,D)** miRNA-mRNA network analysis of miR-1305 and miR-6785-5p, Red and green colours indicated high and low expression, respectively. miRNA: microRNA; EVs: extracellular vesicles. miRna-mRNA net-work analysis was mapped using Cytoscape 3.9.0 software.

To explore the potential functions of differentially expressed miRNAs in serum EVs, we predicted the target genes of the validated miR-1305 and miR-6785-5p using bioinformatics analysis (TargetScanHuman 7.1 and miRDB-MicroRNA Target Prediction Database). As miR-1305 was downregulated and miR-6785-5p was upregulated in serum EVs and psoriatic skin lesions, the predicted target genes of miR-1305 and miR-6785-5p were intersected with upregulated and downregulated mRNAs which were detected by mRNA microarray (GSE181318), respectively. The results showed that 77 upregulated mRNAs might be regulated by miR-1305 and 83 downregulated mRNAs might be regulated by miR-6785-5p (Figures 4A,B); Supplementary Tables S3, S4). In addition, we mapped the miRNA-mRNA networks of miR-1305 and miR-6785-5p to study their regulatory mechanisms with the 77 upregulated mRNAs and 83 downregulated mRNAs, respectively (Figures 4C,D); Supplementary Tables S5,S6).

To clarify the potential biological functions of the 77 upregulated mRNAs and 83 downregulated mRNAs and the predicted involved signalling pathways, we annotated each gene of the intersection based on the Gene Ontology and KEGG database. Then, GO analysis and KEGG pathway analysis were performed to predict the function of miR-1305 and miR-6785-5p, respectively. The results showed that both miR-1305 and miR-6785-5p were involved in regulating the inflammation response and the signalling pathway related to psoriasis, including positive regulating of type 1 interferon production, autophagy, cytokine-cytokine receptor interaction, Th17 cell differentiation, IL-17 receptor activity, Wnt signalling pathway, MAPK signalling pathway and so on (Supplementary Figures S1A,D). These results indicated that miR-1305 and miR-6785-5p might be involved in the pathogenesis of psoriasis.

### Serum extracellular vesicles promote keratinocytes proliferation and inflammation response

**FIGURE 5:**
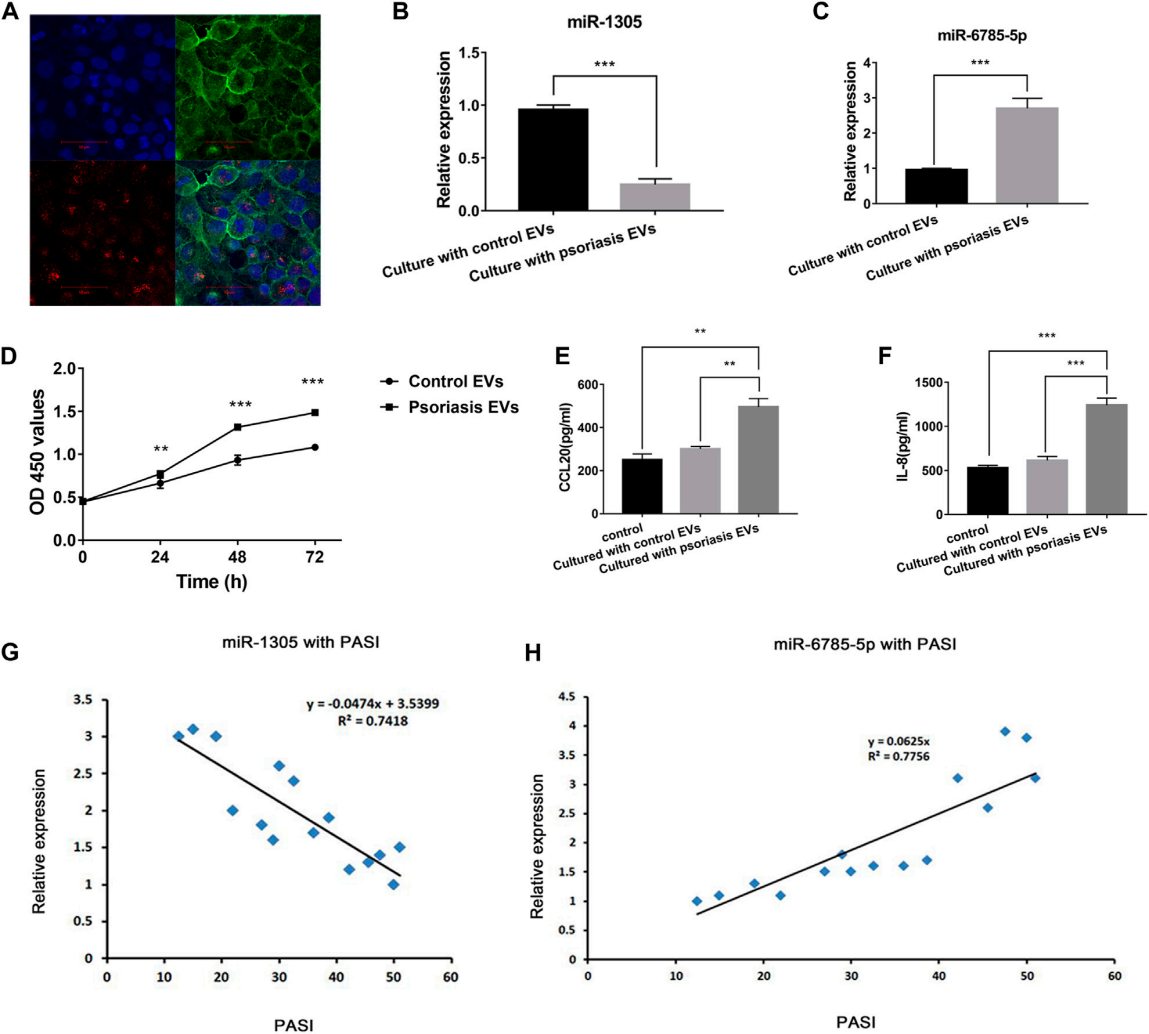
Serum EVs-keratinocytes interaction and serum EVs miR-1305 and miR-6785-5p might be biomarkers of psoriasis. **(A)** Uptake experiment of serum EVs; PKH26-labeled hucMSCs-Exo (red), cytoskeleton was labelled with phalloidin stain (green), and nuclei were stained with DAPI (blue). Scale bar = 50 µm; EVs: extracellular vesicles. **(B,C)** The expression of miR-1305 and miR6785-5p in NHEKs after co-cultured with serum EVs from psoriasis patients and controls. **(D)** The effects of serum EVs from psoriasis patients and controls on the proliferation of NHEKs. OD450: optical density measured at 450 nm. **(E,F)** The effects of serum EVs from psoriasis patients and controls on the secretion of CCL20 and IL-8 by NHEKs. **(G,H)** The expression of serum EVs miR-1305 and miR-6785-5p with PASI. miRNA: microRNA, PASI: plaques are graded based on three criteria: redness (R), thickness (T), and scaliness (S). Severity is rated for each index on a 0–4 scale (0 for no involvement; 4 for severe involvement). The body is divided into 4 regions: head (h), upper extremities (u), trunk (t), and lower extremities (l). In each of these areas, the fraction of total surface area affected is graded on a 0–6 scale (0, no involvement; up to 6 for >90% involvement). The various body regions are weighted to reflect their respective proportion of body surface area. The composite PASI score can then be calculated: PASI = 0.1(Rh + Th + Sh)Ah + 0.2(Ru + Tu + Su)Au + 0.3(Rt + Tt + St)At + 0.4(Rl + Tl + Sl)Al.

To investigate whether serum EVs were involved in the activation of keratinocytes, we performed a serum EVs-keratinocytes interaction experiment. Purified serum EVs were isolated and labelled with PKH26 Red Fluorescent. Next, the labelled EVs were co-cultured with NHEK cells for 12 h. Then, the cells were fixed with 4% formaldehyde and stained with phalloidin-iFluor 488 reagent and DAPI. Using a confocal microscope, we verified that serum EVs could be taken up by NHEK cells (Figure 5A).

Next, we detected the expression of miR-1305 and miR-6785-5p in NHEK cells after co-cultured with serum EVs obtained from psoriasis patients and healthy controls. The results showed that miR-1305 was downregulated and miR-6785-5p was upregulated after co-cultured with serum EVs obtained from psoriasis compared with controls (Figures 5B,C). As the intrinsic cell targeting properties of EVs, our findings assumed that serum EVs might not only directly communicate with neighbouring cells, such as T cells or B cells, but also communicate with the activation of keratinocytes in psoriasis *via* the loaded miRNAs, including miR-1305 and miR-6785-5p.

Then, we explored the role of serum EVs in keratinocyte proliferation and inflammatory response. CCK-8 assay showed that serum EVs obtained from psoriasis significantly promoted the proliferation of keratinocytes compared with that of control groups (Figure 5D). Similarly, the results of ELISA analysis showed the secretion of CCL20 and IL-8 was significantly increased compared with that of control groups (Figures 5E,F). These findings suggested that serum EVs might be involved in the activation of keratinocytes in psoriasis.

Serum EVs miR-1305 and miR-6785-5p are related to the disease severity and might be biomarkers of psoriasis.

To further confirm the relationship of serum EVs miR-1305 and miR-6785-5p with the severity of psoriasis, we measured the correlation of miR-1305 and miR-6785-5p in serum EVs with PASI scores. As Figures 5G,H showed, the increase of miR-6785-5p was positively correlated with the PASI score. In contrast, the expression of miR-1305 in serum EVs was negatively correlated with disease severity. These results indicated that serum EVs miR-1305 and miR-6785-5p might serve as biomarkers of psoriasis.

## Discussion

It is well known that a complex interplay among keratinocytes, immune cells and inflammatory mediators contributes to the pathogenesis of psoriasis (Raeber et al., 2018; Rendon and Schakel, 2019). In recent years, EVs have become a subject of intense study. EVs, such as microvesicles and exosomes, act as cell-to-cell communication vectors and potential biomarkers for diseases (Liu et al., 2019). The impacts of EVs transfer have been reported in many critical cellular processes including cell-to-cell communication and immune response regulation (Robbins et al., 2016). EVs are released by donor cells and can be taken up by recipient cells. EVs can carry RNAs and proteins which may affect the phenotype of the recipient cells (Mulcahy et al., 2014). Although the role of secreted cytokines and chemokines has been well documented in psoriasis, the potential role of EVs has not been sufficiently investigated in this disease (Deng et al., 2016; Yan et al., 2019). The communication between serum EVs and keratinocytes remains less known. Here, we found keratinocytes could take up serum EVs. In addition, considering the intrinsic cell targeting properties of EVs, we assumed that serum EVs might be taken up by keratinocytes and serum EVs loaded miRNAs might be involved in regulating the mRNAs expression and activation of keratinocytes in psoriasis.

EVs are cell-secreted lipid bilayer membranous particles with heterogeneous size and composition (Li et al., 2020). They derive either from the endosomal compartment (exosomes) or as a result of shedding from the plasma membrane (microvesicles, endosomes and apoptotic bodies) (O'Brien et al., 2020). EVs may collapse during drying, resulting in a cup-shaped morphology (Raposo and Stoorvogel, 2013). The average diameter of EVs ranges from nanometres to a few micrometres, and the variety of their biological content includes lipids, proteins, and RNAs (Fabbiano et al., 2020). The major proteins involved with EVs biogenesis include CD63, CD81, CD9, etc (Veziroglu and Mias, 2020). Here in our study, using the methods of TEM and NTA, we verified that purified serum EVs exhibited a typical cup-shaped structure and the mean diameters were around 100 nm. In addition, the protein markers CD9, CD63, and CD81 were detected in serum EVs and the results were consistent with previous studies.

MiRNAs are important gene regulatory molecules and regulate vital cellular processes and inflammation regulation (Fabian et al., 2010). They modulate protein expression by inhibiting mRNA translation and stability. Our previous studies have verified that differentially expressed miRNAs in skin lesions were related to psoriasis, including miR-145-5p, miR-20a-3p, and miR-548a-3p (Li et al., 2018; Zhao et al., 2018; Yan et al., 2019). Recently, EVs miRNAs are considered to be involved in the pathogenesis of psoriasis. A previous study has identified that small EVs containing miR-381-3p from keratinocytes promotes Th1/Th17 polarization in psoriasis (Jiang et al., 2021). Another report identified circulating miRNAs in EVs as potential biomarkers for psoriatic arthritis in patients with psoriasis (Pasquali et al., 2020). However, the role of serum EVs in the pathogenesis of psoriasis remains less known.

Here, we found that miRNAs are indeed abnormally expressed in serum EVs in psoriasis. In addition, we confirmed that keratinocytes could take up the serum EVs. The result laid the foundation for studying the role of serum EVs in regulating the activation of keratinocytes and skin inflammation. As the intrinsic cell targeting properties of EVs, the results of miRNA microarray results and the EVs-keratinocytes interaction experiment indicated that there might be interactions between the dysregulated miRNAs derived from EVs and mRNAs in skin lesions. Furthermore, through the bioinformatics analysis, we found the dysregulated miRNAs derived from EVs might be involved in the activation of keratinocytes in psoriasis.

To further study the role of differential expression miRNAs derived from EVs in psoriasis, we found that miR-1305 and miR-6785-5p were closely related to inflammation response. Previous studies have reported that miR-1305 and miR-6785-5p play important regulating roles in several diseases (Yu et al., 2019; Liu et al., 2020; Su et al., 2020). However, their functions in psoriasis have not been studied before. In this study, we found that miR-1305 and miR-6785-5p were participating in regulating inflammation response associated with psoriasis. Furthermore, the results of the bioinformatic analysis suggested that miR-1305 and miR-6785-5p might be involved in regulating the activation and differentiation of Th17 and Th1, which were not reported before. In addition, our findings indicated that miR-1305 and miR-6785-5pmight play an important role in the pathogenesis of psoriasis (Chang et al., 2018; Roy et al., 2018; Wu et al., 2018).

It is generally accepted that EVs can be taken up and impact the functions of recipient cells. miRNAs in extracellular vesicles (EVs), may mediate paracrine and endocrine communication between different tissues and thus modulate gene expression and the function of distal cells (Mori et al., 2019). Here, we confirmed serum EVs regulated the expression of miR-1305 and miR-6785-5p in NHEKs and promoted NHEKs proliferation and proinflammatory cytokines secretion, including CCL20 and IL-8. CCL20 plays an important role in psoriasis by recruiting CCR6+Th17 cells into the lesional skin (Furue et al., 2020). IL-8 is also an important factor in psoriasis, which is characterized by proliferation of keratinocytes, neutrophil infiltration and angiogenesis (Wu et al., 2017). These results indicated that psoriasis serum EVs were involved in the activation of keratinocytes and skin inflammation in psoriasis. Furthermore, the functions of serum EVs may be through loading and transferring miR-1305 and miR-6785-5p.

Increasing studies have focused on extracellular miRNAs as potential biomarkers, since they are stable and can be detected in the blood, urine, or other body fluids (Grasedieck et al., 2012; Mori et al., 2019). In this study, by analyzing the correlation between the expression of serum EVs miR-1305 and miR-6785-5p with the Psoriasis Area and Severity Index (PASI) score, we found the expression of serum EVs miR-6785-5p was positively correlated with the PASI score. In contrast, the expression of serum EVs miR-1305 was negatively correlated with the PASI score. These results indicated that serum EVs miR-1305 and miR-6785-5p can be served as biomarkers in psoriasis. In addition, miR-1305 and miR-6785-5p may be potential candidates for therapy of psoriasis in the future.

However, this study also has some limitations. Firstly, the tissue sample sizes were relatively small, and larger samples are required in the future to verify our findings. Secondly, the biomarkers only focused on psoriasis. The specificity of the biomarkers miR-1305 and miR-6785-5p in other inflammatory diseases should be investigated. Thirdly, the molecular mechanism of miR-1305 and miR-6785-5p in psoriasis has not yet been fully revealed in this study, and the remaining 14 miRNAs were left out in this study, which limited the investigation of their functions in this study. Finally, due to limitation of the sample size, we only showed the correlation of miR-1305 and miR-6785-5p in serum EVs with PASI scores.

## Conclusion

In conclusion, the current study may suggest that serum EVs might be involved in the activation of keratinocytes through loaded miRNAs in psoriasis. Serum EVs miR-1305 and miR-6785-5p may be associated with psoriasis. This study may provide insight for further into the development and treatment of psoriasis.

## Data Availability

The original contributions presented in the study are publicly available. This data can be found here: GSE200637 and GSE181318.
