# Supplementary material for: Downregulation of miRNA miR-1305 and upregulation of miRNA miR-6785-5p may be associated with psoriasis
Source: Front Genet. 2022 Aug 10;13:891465. doi: 10.3389/fgene.2022.891465 (PMC9399421; doi:10.3389/fgene.2022.891465)
Supplement: Supplementary file 1 [file Table1.DOCX]

| miRNA Name | p-values | foldchange |
| --- | --- | --- |
| hsa-miR-1305 | 0.017111 | 0.238474682 |
| hsa-miR-1914-3p | 0.047641 | 0.334404879 |
| hsa-miR-3127-5p | 0.021371 | 0.200781381 |
| hsa-miR-3198 | 0.015254 | 0.290583616 |
| hsa-miR-4713-3p | 0.031175 | 0.327424311 |
| hsa-miR-5088-5p | 0.049423 | 0.27168316 |
| hsa-miR-5194 | 0.042253 | 0.186328589 |
| hsa-miR-5581-5p | 0.039716 | 0.325887686 |
| hsa-miR-6131 | 0.014001 | 0.130972886 |
| hsa-miR-636 | 0.045555 | 0.45452573 |
| hsa-miR-6717-5p | 0.009819 | 0.117532582 |
| hsa-miR-6767-5p | 0.035737 | 0.345923237 |
| hsa-miR-6780b-5p | 0.009855 | 0.161349467 |
| hsa-miR-6785-5p | 0.011691 | 2.041106826 |
| hsa-miR-6875-5p | 0.007655 | 0.121827693 |
| hsa-miR-7107-5p | 0.021568 | 2.125194747 |

Table S1: Differentially expressed miRNAs in EVs from psoriasis compared with healthy control.
