# Supplementary material for: Downregulation of miRNA miR-1305 and upregulation of miRNA miR-6785-5p may be associated with psoriasis
Source: Front Genet. 2022 Aug 10;13:891465. doi: 10.3389/fgene.2022.891465 (PMC9399421; doi:10.3389/fgene.2022.891465)
Supplement: Supplementary file 2 [file Table7.DOCX]

#### shareable link of the raw data

https://www.jianguoyun.com/p/DaaftlQQ1M2wChiQx7ME
