# Supplementary figures and images for: Downregulation of miRNA miR-1305 and upregulation of miRNA miR-6785-5p may be associated with psoriasis
Source: Front Genet. 2022 Aug 10;13:891465. doi: 10.3389/fgene.2022.891465 (PMC9399421; doi:10.3389/fgene.2022.891465)

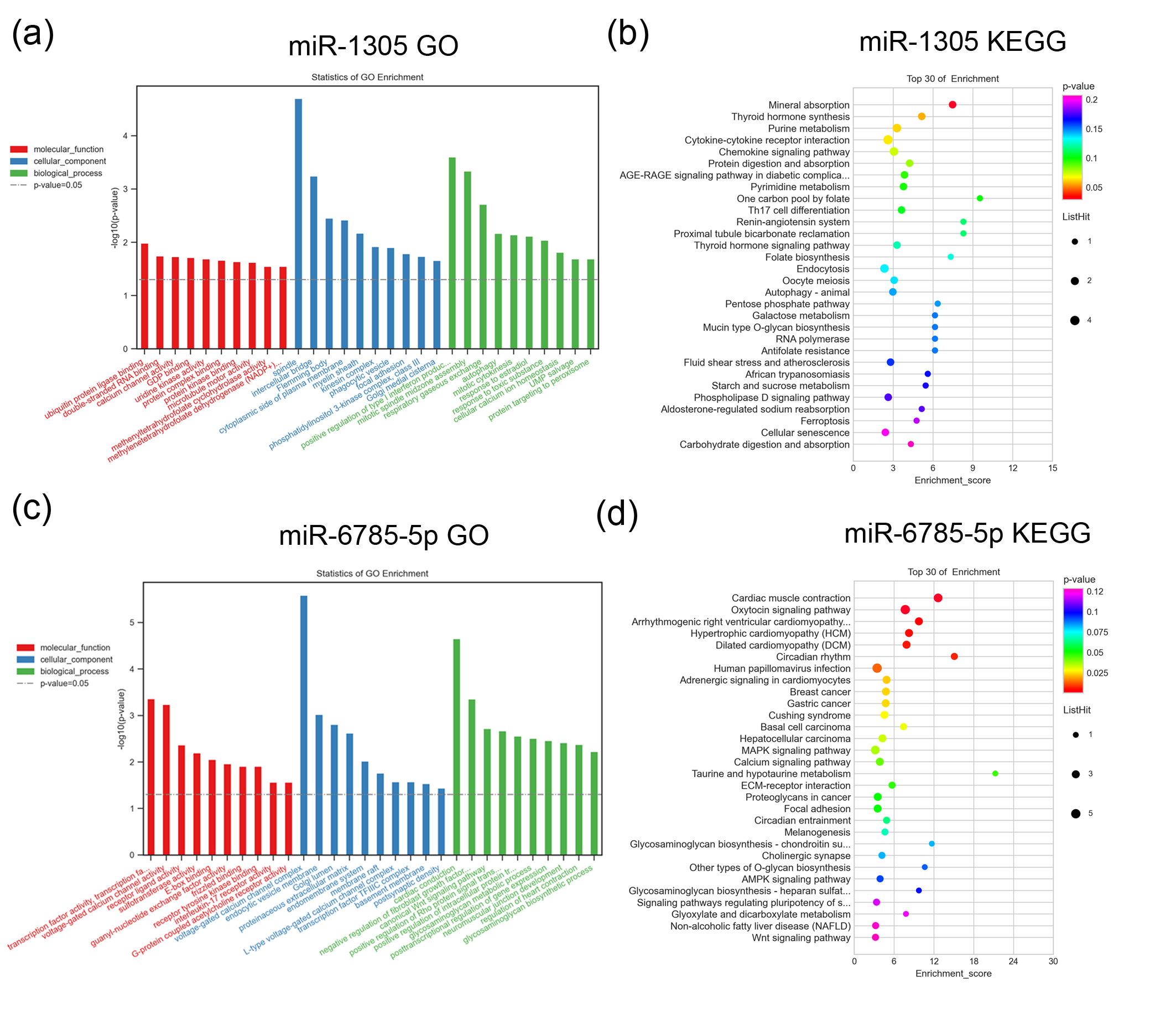

Supplement: Supplementary file 3 [file Image1.TIF]
