# Supplementary material for: Downregulation of miRNA miR-1305 and upregulation of miRNA miR-6785-5p may be associated with psoriasis
Source: Front Genet. 2022 Aug 10;13:891465. doi: 10.3389/fgene.2022.891465 (PMC9399421; doi:10.3389/fgene.2022.891465)
Supplement: Supplementary file 4 [file Table2.DOCX]

Table S2: Result of mRNA microarray analysis in psoriatic skin lesions and healthy control.

| **mRNA** | **pvalues** | **foldchange** |
| --- | --- | --- |
| PGLYRP4 | 0.020195768 | 3.264704836 |
| MGAT3 | 0.033606629 | 0.471528954 |
| C1orf210 | 0.047553958 | 2.107213611 |
| S100A7L2 | 0.008498093 | 116.0884144 |
| DTYMK | 0.000924166 | 3.15152624 |
| SLC25A13 | 0.002766862 | 2.036530721 |
| ZCWPW1 | 0.014078457 | 0.414317935 |
| CD58 | 0.011358787 | 2.107046172 |
| CKM | 0.002547745 | 2.23979169 |
| TAF1B | 0.017677971 | 2.204860748 |
| IFI16 | 0.001667838 | 2.865122616 |
| SKA1 | 0.039268504 | 6.526716693 |
| DOK2 | 0.032673879 | 3.426674318 |
| BRIP1 | 0.012441913 | 2.510746961 |
| KCNK7 | 0.003740917 | 0.470756309 |
| AUNIP | 0.008403582 | 5.065882956 |
| LILRA5 | 0.001212505 | 7.683731228 |
| VSIG10L | 0.001741036 | 3.637488109 |
| TRIP13 | 0.024129924 | 2.818688695 |
| HJURP | 0.030847413 | 3.614141989 |
| ANKDD1B | 0.033354131 | 0.42730506 |
| IL36RN | 0.001954584 | 8.396118048 |
| CYP2W1 | 0.006887066 | 0.04697741 |
| TLE3 | 0.038146672 | 2.131102362 |
| MTFP1 | 0.002937008 | 2.342945868 |
| LCE3E | 0.002604104 | 72.26566557 |
| S100A12 | 0.000287208 | 582.6799629 |
| SRPK3 | 0.009992902 | 0.162353998 |
| MAD2L2 | 0.00027072 | 2.106816471 |
| UTP3 | 0.012798529 | 2.11113081 |
| CXCL3 | 0.021000533 | 3.278005999 |
| HELZ2 | 0.000763221 | 4.013479457 |
| AK9 | 0.010121855 | 0.287760851 |
| ZNF536 | 0.00077942 | 0.243180866 |
| OAS1 | 0.028296157 | 3.976355334 |
| DIAPH3 | 0.00175914 | 2.250622167 |
| CCL8 | 0.009337501 | 6.869867358 |
| MT4 | 0.040947237 | 0.032208206 |
| ARPC1B | 0.008369239 | 2.51658264 |
| MCOLN3 | 0.041331035 | 0.405702027 |
| TNNI2 | 0.002772294 | 0.140989904 |
| ICAM1 | 0.028508214 | 2.205780253 |
| ULBP1 | 0.023807228 | 3.178286486 |
| IRS2 | 0.001285571 | 0.365142318 |
| TYSND1 | 0.018078833 | 0.473794992 |
| PYGL | 0.002198754 | 2.495598625 |
| EDIL3 | 0.014658469 | 0.280605836 |
| SESN2 | 0.012527547 | 2.203984783 |
| VKORC1 | 0.022791053 | 2.040128739 |
| NETO2 | 0.010363503 | 4.332416358 |
| CAMSAP3 | 0.011951106 | 0.465532396 |
| RAI14 | 0.009841464 | 0.422967628 |
| NUDT5 | 0.000323201 | 2.114424162 |
| MLXIP | 0.003623993 | 0.455834197 |
| SFT2D1 | 0.003992354 | 2.492951888 |
| HAVCR2 | 0.0311597 | 2.810240295 |
| FBXO10 | 0.014673892 | 2.116239726 |
| PTPN22 | 0.003327404 | 3.123327987 |
| NISCH | 0.006901725 | 0.471758932 |
| NMI | 0.000872849 | 4.108664669 |
| C9orf84 | 0.011001338 | 0.362561364 |
| GPIHBP1 | 0.015691617 | 0.280083331 |
| ATP6V1G1 | 0.010766372 | 4.418645258 |
| TANGO6 | 0.003550286 | 2.747209282 |
| E2F1 | 0.021159977 | 0.414598634 |
| MAN2A1 | 0.002970586 | 2.065114332 |
| MYO1G | 0.037321313 | 2.933887328 |
| LRP5 | 0.024610231 | 0.489238129 |
| BCAR3 | 0.000861831 | 0.428479572 |
| NDFIP2 | 0.018942378 | 0.468008897 |
| MPDU1 | 0.006530681 | 2.177737929 |
| WNT4 | 0.018287607 | 0.475029878 |
| APOL1 | 0.012451633 | 3.964132104 |
| TNF | 0.005580887 | 2.669146087 |
| FAM171B | 0.024274787 | 0.348089619 |
| MYCL | 0.021514557 | 0.462851267 |
| EMX2 | 0.044333083 | 0.431882394 |
| SPACA4 | 0.00287967 | 2.787977423 |
| CFB | 0.001511019 | 6.362636787 |
| CRHR1 | 0.037700875 | 0.42628338 |
| ZNF83 | 0.041563522 | 0.382097248 |
| NFE2L3 | 0.001398625 | 2.670478065 |
| NME3 | 0.007278159 | 0.471322738 |
| HSD17B13 | 0.026821937 | 0.231567758 |
| MMP1 | 0.002770111 | 25.46921196 |
| CAMP | 0.002238022 | 27.76815693 |
| LRRC59 | 0.014093686 | 2.778907228 |
| PHEX | 0.026678053 | 3.642881441 |
| PER1 | 0.008466756 | 0.210939677 |
| ALS2CL | 0.031706172 | 0.373879634 |
| CD7 | 0.010205692 | 2.568499949 |
| SESN1 | 0.0078106 | 0.462563588 |
| PPM1J | 0.04616845 | 0.458034942 |
| CSRNP3 | 0.021014618 | 0.492924016 |
| NBPF1 | 0.004340005 | 0.458489237 |
| KRT6C | 0.007915332 | 2.114500079 |
| PARD3 | 0.00159845 | 0.353686889 |
| ACTRT3 | 0.033787039 | 4.037768214 |
| PHLDA2 | 0.022761311 | 4.117016026 |
| TTK | 0.001313558 | 3.170643374 |
| FAM26F | 0.012752832 | 5.255537009 |
| BRCA2 | 0.015711286 | 2.419891995 |
| DSC2 | 0.009231751 | 4.077653071 |
| MRTO4 | 0.010355918 | 2.416120213 |
| F12 | 0.000156829 | 3.805269865 |
| SMCO2 | 0.006900128 | 2.554073742 |
| GRAMD1A | 0.020991624 | 2.343009687 |
| GPR1 | 0.002425235 | 5.606001521 |
| SAMSN1 | 0.028405357 | 4.981785131 |
| WAS | 0.017491622 | 2.632274809 |
| LAIR1 | 0.029965911 | 3.534909308 |
| DDX56 | 0.016828793 | 2.128794298 |
| ZNF679 | 0.000376317 | 0.424052669 |
| DDIAS | 0.002741948 | 2.963603728 |
| NAPSA | 0.036973896 | 0.493460973 |
| CCL2 | 0.002498966 | 3.08810897 |
| VWA1 | 0.002273774 | 2.135332216 |
| C1QTNF9B | 0.022684937 | 0.472137355 |
| TRIM25 | 0.013169319 | 2.711020802 |
| NUMA1 | 0.008703703 | 0.481589451 |
| SULT2B1 | 0.010501822 | 2.513761737 |
| CECR1 | 0.032759754 | 2.132293171 |
| TBC1D8 | 0.006712684 | 0.461104197 |
| LAMA5 | 0.002572477 | 0.224464982 |
| GARS | 0.005221256 | 2.810642619 |
| GYG1 | 0.010036108 | 2.968029638 |
| TTC21B | 0.00945149 | 0.383027505 |
| LINC01272 | 0.009241218 | 6.766393265 |
| RPL31 | 0.005487874 | 0.420525209 |
| FBN2 | 0.007938862 | 12.12436478 |
| S100A9 | 0.021313095 | 159.6303428 |
| CXCR3 | 0.042337855 | 5.759209585 |
| ORC6 | 0.002916262 | 2.20945008 |
| LMNB1 | 0.001183897 | 2.948712073 |
| CYP2J2 | 0.010112447 | 0.37474988 |
| LDB1 | 0.037037064 | 0.455552559 |
| CD163 | 0.039885555 | 3.293797206 |
| KLK6 | 0.018810134 | 3.197445507 |
| GLTP | 0.00138593 | 2.142397963 |
| ADAM23 | 0.024899748 | 2.525887107 |
| NKD2 | 0.044554397 | 0.446600806 |
| DOK7 | 0.03963855 | 0.21136744 |
| NFKBIE | 0.015581818 | 2.028180952 |
| SDF2L1 | 0.00044198 | 3.559963521 |
| ZNF273 | 0.00584213 | 0.494258056 |
| BRCA1 | 0.001342554 | 2.140771399 |
| SNAPIN | 0.000114958 | 2.159953494 |
| TRIM14 | 0.006457101 | 4.560209447 |
| TMEM8B | 0.046861189 | 0.445901308 |
| TNFSF10 | 0.010152087 | 2.852393846 |
| CDC25A | 0.012033405 | 4.072038349 |
| APOL6 | 0.005766053 | 5.928407295 |
| TEF | 0.001045215 | 0.393229566 |
| FAM159A | 0.016929036 | 2.40546568 |
| NR1D1 | 0.00803291 | 0.44454661 |
| AMY1A | 0.006139745 | 0.251993498 |
| DPH3P1 | 0.003683944 | 3.392059629 |
| RSPH14 | 0.009656851 | 0.30971534 |
| GOLGA6D | 0.002102461 | 0.482600546 |
| CRABP2 | 0.000770111 | 4.583422003 |
| MESP1 | 0.019554251 | 3.822227633 |
| CTHRC1 | 0.009081111 | 3.261679352 |
| STAR | 0.013379565 | 0.403433048 |
| PBK | 0.00074794 | 3.295835474 |
| TG | 0.032433044 | 0.224381559 |
| TMPRSS11D | 0.001681255 | 40.47278739 |
| S100A16 | 0.004614603 | 2.861739613 |
| FAM162A | 0.017652341 | 2.223142326 |
| TOM1L2 | 0.002437184 | 0.431201816 |
| COX6B2 | 0.004187708 | 0.320843614 |
| PLD6 | 0.026948222 | 0.451997659 |
| MICALL1 | 0.001126369 | 2.684573242 |
| C1orf177 | 0.044981627 | 2.597656073 |
| USB1 | 0.003895061 | 6.41957648 |
| PRKDC | 0.001488915 | 2.288643624 |
| GLIS1 | 0.031941593 | 0.321962701 |
| CACNA1H | 0.031872967 | 0.045727886 |
| DHRS13 | 0.012545908 | 2.074114799 |
| CD274 | 0.002115714 | 15.38668632 |
| CASP7 | 0.045983504 | 2.351728247 |
| CCDC3 | 0.016146532 | 0.416710869 |
| SERPINB13 | 0.001497531 | 5.899401909 |
| LMNB2 | 0.007142091 | 2.469288063 |
| GLB1L3 | 0.02797141 | 3.658516351 |
| DNAJB4 | 0.003006616 | 0.406715864 |
| MID1 | 0.002551004 | 2.51456577 |
| SPRR2G | 0.004197198 | 162.2352536 |
| PRSS56 | 0.001955102 | 0.383780906 |
| POLQ | 0.000260204 | 2.041729529 |
| S1PR4 | 0.047079885 | 3.509314216 |
| ZNF211 | 0.008243172 | 0.498122792 |
| DERL3 | 0.009083971 | 2.078249507 |
| EFCAB6 | 0.00484096 | 0.326819789 |
| ELAVL1 | 0.012353639 | 0.482522519 |
| COL6A3 | 0.026650505 | 2.097342242 |
| CNTNAP3 | 0.009551976 | 0.33916085 |
| NEU2 | 0.00496967 | 5.630027547 |
| PROK2 | 0.013190871 | 6.681364841 |
| LILRB4 | 0.030441108 | 4.604089459 |
| KLK10 | 0.002826736 | 6.306666156 |
| IFI27 | 0.004877407 | 7.782985302 |
| ZNF254 | 0.005155297 | 0.36766541 |
| FST | 0.026637093 | 0.343207993 |
| CD300LB | 0.017506864 | 3.570073124 |
| CD1A | 0.012314197 | 0.440171176 |
| CTRB2 | 0.000114673 | 7.262171437 |
| TCERG1L | 0.049200936 | 3.14788376 |
| SOD2 | 0.008006554 | 5.574021098 |
| NKG7 | 0.043212446 | 7.338739344 |
| LRIT2 | 0.030425737 | 0.477491495 |
| SERTAD1 | 0.008540879 | 2.035856715 |
| PI3 | 0.003145301 | 199.5888663 |
| TXNIP | 0.01496673 | 0.397720417 |
| CD36 | 0.028870438 | 3.627103804 |
| FAM89A | 0.004358249 | 2.129631779 |
| SNX10 | 0.02378128 | 5.163879279 |
| CENPA | 0.010341284 | 2.130521961 |
| E2F8 | 0.012368651 | 2.159497625 |
| ANKRD22 | 0.002009112 | 2.113199916 |
| HOTS | 0.02961235 | 0.214634978 |
| MST1L | 0.014512628 | 0.245226022 |
| OLFM1 | 0.025594743 | 2.350684252 |
| DDX58 | 0.003085197 | 4.639446749 |
| SLC34A1 | 0.007577233 | 2.918932039 |
| LOC728485 | 0.033103682 | 0.325506908 |
| MREG | 0.000766061 | 3.463393147 |
| C22orf15 | 0.024435782 | 0.35650608 |
| ACTL10 | 0.000861903 | 2.559964771 |
| ESPN | 0.004587589 | 0.389027196 |
| TNNT3 | 0.026406863 | 0.40559116 |
| TSC22D3 | 0.014233666 | 0.331808869 |
| CHADL | 0.005134715 | 0.270853042 |
| IFI35 | 0.013003787 | 2.552841183 |
| ACADL | 0.022562704 | 0.234971217 |
| ARNTL2 | 0.004475815 | 3.0122472 |
| FOLH1B | 0.039654083 | 3.895427051 |
| STARD4 | 0.043811527 | 3.100810288 |
| PER3 | 0.043925507 | 0.440229373 |
| HYAL3 | 0.014241378 | 2.855513396 |
| CXCR6 | 0.021303846 | 5.775317103 |
| PKIB | 0.03334828 | 0.449388916 |
| PAFAH2 | 0.012866956 | 0.486046641 |
| AMMECR1 | 0.011295001 | 2.719598932 |
| BOC | 0.000312046 | 0.287497117 |
| CENPW | 0.005398401 | 2.455560839 |
| SNRPF | 0.029153584 | 2.344633524 |
| COA6 | 0.005149122 | 2.308622097 |
| PNO1 | 0.000241606 | 3.637263192 |
| TSNARE1 | 0.000844501 | 0.282647966 |
| PRELID1 | 0.001273118 | 2.716924382 |
| EPHB6 | 0.014986196 | 0.392314095 |
| SPTBN5 | 0.037562192 | 0.405514783 |
| SEC31B | 0.024019644 | 0.416058699 |
| APELA | 0.006027636 | 2.93686026 |
| TEKT3 | 0.001332902 | 0.312534541 |
| TGIF2-C20orf24 | 0.008799718 | 3.018147385 |
| KIF18A | 0.003909195 | 3.660041794 |
| CFAP57 | 0.034500852 | 0.443044564 |
| SLC6A14 | 0.002260363 | 34.92193828 |
| ITPRIPL1 | 0.010132705 | 2.058420781 |
| NBPF11 | 0.001610652 | 0.428583593 |
| LPCAT1 | 0.000599969 | 2.602279626 |
| TICRR | 0.008467827 | 3.093222826 |
| TUBB6 | 0.000695404 | 2.72787093 |
| HNRNPAB | 0.008735575 | 2.178583189 |
| NANS | 0.009283508 | 2.149596301 |
| EXPH5 | 0.049972101 | 0.428953965 |
| PCP2 | 0.007628177 | 0.392718731 |
| FBXW11 | 0.007587091 | 2.24689023 |
| LRRC25 | 0.005299683 | 8.381318748 |
| GATA3 | 3.66434E-06 | 0.245667628 |
| VCAN | 0.0481151 | 2.058538008 |
| EPGN | 0.002312891 | 28.90886052 |
| THRA | 0.001147315 | 0.458505259 |
| C12orf29 | 0.017427361 | 2.849903929 |
| CD300E | 0.00223671 | 7.604590882 |
| ZNF395 | 0.011948695 | 0.461931096 |
| CCDC109B | 0.000115613 | 4.946610716 |
| SPC24 | 0.014964324 | 2.518435969 |
| GCH1 | 0.003983114 | 2.872882868 |
| POLR2I | 0.003186528 | 3.533425381 |
| NLRX1 | 0.00364656 | 2.533906444 |
| CDC20 | 0.003033317 | 2.720346801 |
| RAB3B | 0.002753688 | 0.154746573 |
| ILF2 | 0.005281423 | 2.077856937 |
| NDUFA9 | 0.002098707 | 2.384197164 |
| LGALS9B | 0.000639004 | 4.381586194 |
| PITPNM1 | 0.021012923 | 2.300804709 |
| REC8 | 0.024285749 | 2.064121024 |
| PYCARD | 0.04462964 | 2.110135907 |
| ACSF2 | 0.022360835 | 0.403876168 |
| COBL | 0.007471835 | 0.410866982 |
| FAM157A | 0.002550204 | 7.519387124 |
| VSNL1 | 0.01329279 | 2.786635961 |
| SLN | 0.034670426 | 0.123914585 |
| NTPCR | 0.007357934 | 2.439095549 |
| SNRPG | 0.00507304 | 2.378106166 |
| KLHDC7B | 0.007230506 | 21.46847488 |
| BIK | 0.002504875 | 2.446199641 |
| DCAF13 | 0.006957523 | 2.261943239 |
| CLDN1 | 0.0001169 | 0.274628925 |
| IFIT3 | 0.009937304 | 10.1477159 |
| MAL | 0.029749279 | 0.429002286 |
| MAD2L1 | 0.003616265 | 3.3088426 |
| CDX1 | 0.044066405 | 2.23636841 |
| FOSL1 | 0.00390725 | 15.35150313 |
| SQLE | 0.035733888 | 2.84798071 |
| CCR2 | 0.012896869 | 3.685950531 |
| ACO2 | 0.026982307 | 2.05900732 |
| KLK9 | 0.001215286 | 11.814119 |
| ZNF100 | 0.009732284 | 0.497997507 |
| TYMS | 0.010752156 | 2.058190358 |
| LRRC17 | 0.027142658 | 0.438217455 |
| GAS6 | 0.015897745 | 0.47665768 |
| CASC5 | 0.02935331 | 2.918638117 |
| TONSL | 0.00589128 | 2.048742536 |
| TMPRSS7 | 0.012099169 | 0.352884161 |
| TPX2 | 0.002436665 | 2.157409978 |
| APOBEC3A | 0.001059192 | 81.24067317 |
| C9orf91 | 0.01456925 | 2.174511699 |
| EFNB3 | 0.01409021 | 0.348459208 |
| PTTG1 | 0.001898658 | 3.220628826 |
| ADAMTS5 | 0.027100283 | 2.064317771 |
| DNASE1L3 | 1.5892E-05 | 10.91631891 |
| HIST1H3J | 0.042196908 | 4.287332249 |
| CD22 | 0.004779526 | 3.963406571 |
| MELK | 0.006306426 | 4.08479302 |
| THBD | 0.000781554 | 3.461097767 |
| XPO5 | 0.014100536 | 2.06019992 |
| PPIL1 | 0.003573325 | 3.312372403 |
| MRPL50 | 0.012984791 | 2.473685097 |
| DDX60L | 0.002979359 | 2.967246497 |
| APOBEC3C | 0.029751331 | 2.427776764 |
| TNK2 | 0.011158025 | 0.462749365 |
| SEMA5A | 0.020020004 | 0.433496319 |
| CLEC4F | 0.020789923 | 0.286805259 |
| P2RY2 | 0.045544596 | 2.39267174 |
| PLAC8 | 0.032734106 | 8.502149686 |
| PIF1 | 0.001181153 | 2.770750978 |
| INSL3 | 0.036414062 | 2.303105429 |
| HLF | 0.011368226 | 0.384402246 |
| SH2B3 | 0.037392811 | 2.250427082 |
| SOWAHD | 0.006337382 | 2.489321963 |
| APH1A | 0.019028391 | 0.398120516 |
| CARHSP1 | 0.000259722 | 3.505573067 |
| S100A11 | 0.012501011 | 2.323332071 |
| GALR2 | 0.040870985 | 5.551666261 |
| ESYT3 | 0.00753049 | 2.742541361 |
| ADH6 | 0.026554302 | 0.089925493 |
| HIST2H2AB | 0.014387118 | 2.736127155 |
| STEAP1 | 0.045509609 | 2.981918756 |
| HSPB3 | 0.039360081 | 0.306268924 |
| FGD3 | 0.000797984 | 2.429823352 |
| GPATCH4 | 0.007253808 | 2.21992385 |
| PRAMEF22 | 0.001284736 | 0.431663059 |
| IFNA14 | 0.017939843 | 0.206358398 |
| ANXA3 | 0.037456476 | 2.79471292 |
| DNAJB6 | 0.002537943 | 2.176654009 |
| CMTM5 | 0.009831498 | 0.324723044 |
| APCDD1 | 0.003081239 | 0.484892333 |
| MRPL15 | 0.000431497 | 0.437772549 |
| BCL2A1 | 0.010769892 | 2.873487214 |
| C9orf139 | 0.034239359 | 4.15200374 |
| KLK7 | 0.017777064 | 2.404947396 |
| COL4A1 | 0.0126065 | 2.254872205 |
| ATP13A3 | 0.017163361 | 2.101020222 |
| ULBP2 | 0.023691081 | 6.532583331 |
| C1GALT1C1L | 0.02272128 | 4.521526593 |
| ZNF208 | 0.006521861 | 0.366314571 |
| SCO2 | 0.002717931 | 4.297529649 |
| PLSCR1 | 0.003950421 | 4.633953889 |
| VNN3 | 0.001232919 | 127.9692463 |
| TARS | 0.006459511 | 2.521138128 |
| EPN3 | 0.025186657 | 2.195269843 |
| CDC45 | 0.00930727 | 2.766227994 |
| HERC6 | 0.000654487 | 20.07878095 |
| FCHO1 | 0.037947181 | 0.49445702 |
| EIF4A1 | 0.001416224 | 2.181398656 |
| TMEM45B | 0.022073922 | 3.339408377 |
| CTPS1 | 0.000611673 | 3.634304057 |
| HSPA8 | 0.009774985 | 2.27084977 |
| CNKSR2 | 0.003151159 | 0.363992861 |
| TCAF2 | 0.000489037 | 4.012859057 |
| TAF5L | 0.044813654 | 2.026014707 |
| CATSPER1 | 0.002755308 | 2.88342468 |
| NPIPA1 | 0.023789211 | 0.383373478 |
| APOBEC3D | 0.048518548 | 2.150955216 |
| RUVBL1 | 0.001005046 | 2.290353321 |
| SNRNP25 | 0.011645138 | 3.152202564 |
| ADAM19 | 0.039771261 | 3.511829775 |
| EAF1 | 0.004049243 | 2.627342093 |
| BTC | 0.001494125 | 0.033272657 |
| SCD | 0.040109277 | 2.671994308 |
| MATN2 | 0.025895411 | 0.47109121 |
| PHYHIP | 0.019323355 | 0.099915791 |
| PML | 0.000321717 | 2.534768649 |
| RHOBTB3 | 0.020165409 | 0.449525665 |
| TGFB1 | 0.01666685 | 2.365474852 |
| SNRNP40 | 0.000367454 | 2.220198847 |
| RAP2A | 0.016177862 | 2.266896798 |
| TEX101 | 0.008742507 | 8.27901327 |
| CCRL2 | 0.00545599 | 3.008861834 |
| JMJD7-PLA2G4B | 0.044391118 | 2.381370102 |
| PLCB4 | 0.024575056 | 0.484707448 |
| HIST1H3H | 0.001327043 | 3.123134651 |
| SPTLC2 | 0.010964954 | 2.211634252 |
| ZNF626 | 0.007312248 | 0.367833901 |
| CORO1A | 0.043928401 | 2.300535062 |
| PI15 | 0.038508778 | 5.09711896 |
| DUSP1 | 0.026847467 | 0.241888547 |
| FAM83E | 0.032943366 | 0.436649627 |
| ZNF593 | 0.005276504 | 2.077919506 |
| CDCA8 | 0.009772139 | 2.569954296 |
| CLEC7A | 0.016941158 | 4.332514839 |
| VWA3A | 0.039126046 | 2.037684641 |
| PRSS53 | 0.040152052 | 7.443898209 |
| OSM | 0.011024395 | 5.989076044 |
| FAM86B2 | 0.008155858 | 2.019068085 |
| C1QC | 0.00853682 | 4.139834782 |
| ZNF528 | 0.021485826 | 0.414759477 |
| CFAP44 | 0.024280336 | 0.459007012 |
| LAMC3 | 0.03837088 | 0.347563711 |
| CYTH4 | 0.031003234 | 3.066751339 |
| HIRA | 0.006024196 | 4.531884882 |
| PGAM1 | 0.000130469 | 2.575041276 |
| KANK1 | 0.000895076 | 0.454268831 |
| ALG1L2 | 0.004489036 | 2.02511258 |
| DOK3 | 0.000718004 | 3.041664168 |
| TUBG1 | 0.022671669 | 2.177809819 |
| TNFRSF21 | 0.012893707 | 3.808492505 |
| SLC25A19 | 0.045368168 | 2.234949621 |
| ITPA | 0.002446448 | 2.338411621 |
| RNF212 | 0.007275186 | 0.46550616 |
| RASAL3 | 0.046838743 | 2.412423482 |
| DIMT1 | 0.002466235 | 2.297332842 |
| ASIC3 | 0.046765894 | 0.453912122 |
| CMTM4 | 0.005234024 | 0.446778183 |
| RAD51AP1 | 0.006234119 | 2.887458881 |
| HIST1H2AL | 0.006885935 | 3.242621232 |
| TMEM54 | 0.000585285 | 3.667885539 |
| RAB40C | 0.00099053 | 0.4442108 |
| SPC25 | 0.002957986 | 5.253971859 |
| NUAK1 | 0.00377554 | 0.285761211 |
| UHRF1 | 0.010113229 | 2.499983319 |
| LIN7B | 0.012181621 | 0.415414672 |
| SPSB3 | 0.002424189 | 0.403257194 |
| PFN3 | 0.013504717 | 3.379136264 |
| SH2D2A | 0.014253723 | 4.748963222 |
| SMPD3 | 0.005498286 | 2.756318228 |
| PLEKHH1 | 0.032628282 | 0.43529503 |
| NOP56 | 0.004195735 | 2.402203733 |
| KRT6B | 0.038476941 | 8.528035456 |
| PAX9 | 0.048655851 | 3.463770899 |
| GSDMC | 0.013591191 | 2.161333602 |
| FERMT1 | 0.024672334 | 2.532420877 |
| PRDM1 | 0.022128306 | 2.759593132 |
| COL7A1 | 0.009626099 | 0.372256059 |
| TGM1 | 0.001572151 | 4.678600239 |
| BANF1 | 0.00106842 | 3.25456251 |
| RABL2A | 0.002701876 | 0.468181849 |
| CLCA2 | 0.024470662 | 2.020125503 |
| PDE4D | 0.001398429 | 2.044217441 |
| PROCR | 0.028527401 | 2.885471686 |
| ORC1 | 0.016876851 | 2.438059769 |
| C11orf80 | 0.000932255 | 0.389472883 |
| ALOX12B | 0.017336359 | 2.360664755 |
| SNCA | 0.020296807 | 0.485067423 |
| EEF2K | 0.002319874 | 0.32599506 |
| PCYT1A | 0.03744924 | 2.023521257 |
| HTR7 | 0.012822214 | 3.677321094 |
| SDHAF3 | 0.006198838 | 2.430254334 |
| SAMHD1 | 0.01054145 | 3.196478296 |
| FUT10 | 0.000262187 | 0.484971748 |
| C17orf105 | 0.031148793 | 0.366864626 |
| RAET1G | 0.004619756 | 6.452818953 |
| C1orf95 | 0.000648999 | 0.176632863 |
| GALNT13 | 0.005329042 | 4.539161502 |
| MUC5AC | 0.04280961 | 2.16087241 |
| KCNG1 | 0.020596433 | 2.184807046 |
| OAS3 | 0.000258478 | 9.050180557 |
| GRIK2 | 0.046664957 | 0.326335308 |
| ITPR3 | 0.018125009 | 0.482324614 |
| SMIM9 | 0.00194794 | 0.186221853 |
| USP54 | 0.006048434 | 0.442458295 |
| HIST1H3C | 0.006098267 | 0.466454773 |
| HIST1H2AG | 0.011743648 | 2.264548656 |
| UCK2 | 0.00596514 | 3.119843331 |
| FZD9 | 0.040264925 | 2.034248458 |
| LYPD5 | 0.009540719 | 4.155019833 |
| G0S2 | 0.021807596 | 7.762613 |
| EPSTI1 | 0.003131723 | 8.435645763 |
| GTF3C1 | 0.008554485 | 0.480220371 |
| CDCA3 | 0.007680516 | 2.135071603 |
| NABP1 | 0.004663036 | 2.327744355 |
| C9orf62 | 0.001552876 | 6.442161091 |
| HAS3 | 0.003477389 | 7.992095438 |
| MAP6 | 0.017229665 | 0.463875641 |
| CLDN5 | 0.032219546 | 0.363129985 |
| NUPR1 | 0.00376249 | 0.496062993 |
| DACT2 | 0.03001337 | 0.428665171 |
| HPSE | 0.024093224 | 3.93362138 |
| HEATR3 | 0.009595036 | 2.773206233 |
| ZNF727 | 0.011224853 | 0.418761148 |
| CLDN23 | 0.039137882 | 0.232814772 |
| MS4A6A | 0.036113135 | 4.385136417 |
| GSDMB | 0.034438833 | 0.437935284 |
| RALB | 0.03346839 | 2.423144854 |
| RGMB | 0.007384225 | 0.306160501 |
| APOC2 | 0.039968075 | 0.282295249 |
| CENPN | 0.000697017 | 3.85397609 |
| HSH2D | 0.001056541 | 6.377791564 |
| TMEM206 | 0.001591707 | 2.631867148 |
| FLVCR2 | 0.005486569 | 3.843417998 |
| CHRNA6 | 0.02104521 | 2.664659839 |
| ASIP | 0.020458096 | 0.384129432 |
| CTNNBIP1 | 0.014547612 | 0.401085572 |
| LCE2A | 0.024632238 | 2.126638357 |
| ZNF165 | 0.001952437 | 2.191901813 |
| SERPINB8 | 0.006980174 | 2.145421292 |
| KMT2B | 0.008710945 | 0.45407759 |
| CYBB | 0.009230288 | 5.902557063 |
| WFIKKN1 | 0.010231842 | 0.313528512 |
| RELL2 | 0.00116808 | 2.211016344 |
| FAM169A | 0.015297136 | 2.606546917 |
| WFDC12 | 0.014090415 | 12.56159338 |
| PELI2 | 0.017968909 | 0.398280138 |
| SCPEP1 | 0.03831437 | 0.430000429 |
| MCM10 | 0.003263863 | 3.756727829 |
| UBE2M | 0.005903335 | 2.205487694 |
| CCDC64 | 0.028538082 | 0.483144889 |
| RASSF6 | 0.042289649 | 0.451601897 |
| PTGER3 | 0.024391915 | 2.014760757 |
| AACS | 0.018625079 | 0.494066688 |
| TYROBP | 0.033946393 | 2.376608169 |
| SLC35E4 | 0.010497345 | 2.286333071 |
| KIF18B | 0.002517456 | 2.422601074 |
| MXD1 | 0.041043815 | 2.422772126 |
| ATP12A | 0.022973479 | 34.44254043 |
| PITX2 | 0.045441766 | 0.38816357 |
| UGT1A9 | 0.048275668 | 3.747626798 |
| ACKR2 | 0.003397501 | 18.72331137 |
| ZNF573 | 0.003811737 | 0.458239573 |
| AVEN | 0.015721557 | 2.016689767 |
| CXCL6 | 0.032249091 | 19.73149623 |
| SUN1 | 0.005885298 | 0.412457501 |
| RND2 | 0.0381089 | 0.346991717 |
| KLK13 | 0.018260885 | 13.64530934 |
| HIST1H2BF | 0.007408575 | 3.076953093 |
| RTN4RL1 | 0.001283667 | 0.340924753 |
| ITGB1BP1 | 0.039292699 | 2.794986743 |
| CCL3 | 0.034152485 | 7.927820471 |
| ABCA5 | 0.003292626 | 0.38308026 |
| RAD51B | 0.009793633 | 0.403496364 |
| TIMM17A | 0.00744764 | 2.45088597 |
| AGPAT5 | 0.011241145 | 2.489472148 |
| PLA2G4D | 0.001371284 | 10.3567083 |
| CAV2 | 0.03705646 | 0.491489469 |
| SLC35B2 | 0.012963148 | 2.15977209 |
| CCL3L1 | 0.046949237 | 8.695862166 |
| GSTA3 | 0.013739282 | 0.286941466 |
| LAP3 | 0.032227152 | 2.965697828 |
| ATP13A5 | 0.014092223 | 0.446414354 |
| ACVR1B | 0.003383461 | 0.485784967 |
| SLITRK6 | 3.18828E-05 | 0.305860789 |
| RASSF9 | 0.004167773 | 0.431946474 |
| LILRB2 | 0.023626386 | 3.899672445 |
| PLCH2 | 0.003671964 | 0.24370362 |
| XKRX | 0.011485456 | 2.215011322 |
| PIK3C2G | 0.005772205 | 0.440691441 |
| FBXO6 | 0.003959359 | 3.461896329 |
| CDH26 | 0.02532727 | 4.827195681 |
| SOST | 0.00554047 | 19.20306478 |
| HSPA12A | 0.045257251 | 0.440616457 |
| TRAPPC6A | 0.00213647 | 0.331243527 |
| ZDHHC11 | 0.001572153 | 0.200064053 |
| CXorf21 | 0.044350753 | 2.26814729 |
| LYRM9 | 0.033823979 | 0.413295213 |
| NPTX2 | 0.017231128 | 0.383044273 |
| SGSM1 | 0.000526339 | 0.407657041 |
| GINS2 | 0.003698504 | 2.885599136 |
| CLEC9A | 0.045194027 | 4.265447595 |
| CCNYL1 | 0.00854721 | 2.00560367 |
| CCRN4L | 0.023024599 | 4.979911209 |
| C1R | 0.024611323 | 2.49524883 |
| PRRC2B | 0.018218834 | 0.485659093 |
| IL22 | 0.002174951 | 8.426902971 |
| GPR171 | 0.033569092 | 3.159934429 |
| STXBP6 | 0.029230944 | 0.384222869 |
| FUT7 | 0.022044034 | 3.75170696 |
| TUBB3 | 0.026568206 | 3.508557052 |
| AHSA2 | 0.035476248 | 0.456751136 |
| CANX | 0.018321226 | 2.136001903 |
| SFXN1 | 0.025628671 | 2.272123914 |
| UGT3A2 | 0.039549017 | 0.157636881 |
| CYP4Z1 | 0.005569454 | 3.322037226 |
| CRY2 | 0.003424319 | 0.31082061 |
| PCSK9 | 0.000128991 | 7.101596358 |
| TMEM171 | 0.023175641 | 6.804317253 |
| EHD4 | 0.017702038 | 2.294886129 |
| ADH1B | 0.016877724 | 0.454220496 |
| VPS53 | 0.041974772 | 0.33831648 |
| CASP5 | 0.03293876 | 24.74277973 |
| CEP19 | 0.002326821 | 2.137576591 |
| TBATA | 0.01911716 | 0.094361502 |
| ABCG2 | 0.001624542 | 0.230090481 |
| HOMER1 | 0.012088665 | 2.346599839 |
| SPRR2E | 0.015800836 | 12.64378293 |
| TAF13 | 0.022394734 | 2.453680285 |
| PLA2G2F | 0.001295633 | 3.623542009 |
| S100A7A | 0.008402968 | 672.7581571 |
| ISG20 | 0.014509515 | 3.467594003 |
| GPR68 | 0.011857579 | 2.935490547 |
| HMOX2 | 0.007589311 | 2.480002563 |
| SPRR3 | 0.034561168 | 3.180613009 |
| IRF7 | 0.002536822 | 6.335859248 |
| LYZ | 0.020401567 | 5.551723001 |
| UBE2J1 | 0.019177488 | 2.063892181 |
| TRANK1 | 0.021469188 | 2.058813765 |
| SPRR1B | 0.004511619 | 18.9602619 |
| ALDH4A1 | 0.001698096 | 2.406354713 |
| HDAC1 | 0.000120886 | 2.333926422 |
| USP18 | 0.007220021 | 7.090636124 |
| RDH10 | 0.032679549 | 2.19594657 |
| SCRG1 | 0.013421148 | 0.263675737 |
| WDR53 | 0.003963372 | 2.335516095 |
| OVGP1 | 0.029758756 | 0.267748847 |
| HSF4 | 0.009973428 | 0.32774793 |
| RPS6KA4 | 0.002087961 | 2.200731129 |
| SPRR2B | 0.01536182 | 15.38952172 |
| HIST1H2BE | 0.011913151 | 2.765770521 |
| FOXE1 | 0.015941363 | 9.521612368 |
| LGALS9 | 0.006560339 | 2.717736108 |
| GPR17 | 0.010936961 | 0.391211443 |
| CCL4L1 | 0.02284761 | 7.373530881 |
| CCR7 | 0.033603257 | 7.574974664 |
| APOBEC3B | 0.046507485 | 4.022537113 |
| C11orf40 | 0.008553654 | 0.463468848 |
| SERPINA12 | 0.017682208 | 0.265918386 |
| ITGB6 | 0.014085067 | 2.112042818 |
| HIST1H2AI | 0.006432911 | 3.411739374 |
| KIF15 | 0.000232677 | 2.393961207 |
| CD207 | 0.044893791 | 0.345406739 |
| CCNB1 | 0.003101416 | 2.969044563 |
| CEP55 | 0.000437904 | 4.083289235 |
| XBP1 | 0.015503871 | 2.406219969 |
| VPS33A | 0.000660135 | 2.157778717 |
| SPRY2 | 0.01447579 | 0.464250843 |
| TIGD3 | 0.015782121 | 2.266147845 |
| SELL | 0.023410587 | 11.73597776 |
| DDX21 | 0.007324742 | 2.284461277 |
| SPRR2A | 0.014077106 | 22.20313952 |
| CCR5 | 0.026052602 | 5.575744307 |
| MCF2L | 1.99242E-05 | 0.217922259 |
| SHC2 | 0.012889963 | 0.280127773 |
| JADE1 | 0.022862024 | 0.390854478 |
| HIST1H4L | 0.044491115 | 2.358588503 |
| GDA | 0.002105475 | 21.66164759 |
| NCAPH | 0.023622746 | 3.068839156 |
| ITGA4 | 0.021244668 | 2.784937802 |
| LSM12 | 0.001423886 | 2.017479342 |
| PKDREJ | 0.003904036 | 0.346653696 |
| ANKRD36B | 0.003120569 | 0.499255848 |
| LYSMD2 | 0.002443388 | 2.161184342 |
| LGI3 | 0.047095706 | 0.329374086 |
| LGALS9C | 0.010355293 | 2.861842049 |
| ATL1 | 0.002619202 | 0.343582067 |
| TYMSOS | 0.01158868 | 2.986131741 |
| FKBPL | 0.020705546 | 2.030759545 |
| STEAP4 | 0.000579083 | 4.015073645 |
| MYZAP | 0.007543353 | 3.081556592 |
| SEC14L5 | 0.029034685 | 0.403388398 |
| OSMR | 0.005585395 | 2.129029371 |
| HMMR | 0.014604863 | 2.574094843 |
| CST7 | 0.022103381 | 5.187486995 |
| IFITM3 | 0.008982669 | 2.112878859 |
| BAK1 | 0.003070329 | 3.44082944 |
| PGAM4 | 0.002722357 | 2.003210148 |
| CKS2 | 0.006997817 | 3.595088757 |
| APITD1 | 0.000707045 | 2.301984116 |
| ZNF471 | 0.004216877 | 0.32050334 |
| PRLR | 0.031382065 | 0.470635776 |
| RNF222 | 0.007887254 | 3.880481421 |
| SOX6 | 0.005707496 | 0.343921881 |
| MAP4K1 | 0.01673093 | 2.0839113 |
| OPHN1 | 0.001983082 | 0.497355975 |
| RRM2 | 0.005528876 | 4.384560772 |
| ARSA | 0.011039937 | 0.491763882 |
| ALYREF | 0.015903647 | 2.258170647 |
| NAA10 | 0.00050077 | 2.219709772 |
| GTF3C6 | 0.002126909 | 2.437072431 |
| PLGRKT | 0.001688481 | 2.294485246 |
| AMY2A | 0.00386845 | 0.248455462 |
| HIST1H4K | 0.029115087 | 2.02721635 |
| PPIF | 0.003168282 | 3.73685187 |
| TRIM21 | 0.015099486 | 2.662732839 |
| CHAD | 0.000565782 | 0.125499738 |
| AQP5 | 0.026023768 | 13.4796727 |
| CCDC42B | 0.001948927 | 0.32991773 |
| ST6GALNAC1 | 0.039722768 | 3.546865268 |
| GBA | 0.003102816 | 2.376448861 |
| FOXM1 | 0.007147083 | 2.937162804 |
| EDA | 0.003454263 | 0.421569355 |
| CLPX | 0.011221162 | 2.150809963 |
| TIMM8A | 0.017912884 | 2.071014954 |
| OTOA | 0.046796166 | 2.399332805 |
| GNA15 | 0.002890351 | 2.446464881 |
| ADRB2 | 0.03553192 | 0.402700202 |
| SNRNP200 | 0.005284651 | 0.44141374 |
| PRSS3 | 0.004400647 | 5.648286434 |
| NTSR1 | 0.035716151 | 4.731025922 |
| ODF3B | 0.016634891 | 2.432772024 |
| NRBP2 | 0.016454429 | 0.294691257 |
| SLC25A44 | 0.005308042 | 2.07396867 |
| ANKRD62 | 0.000376202 | 31.02228629 |
| ADAP2 | 0.000561264 | 5.222634516 |
| LRRC61 | 0.001976085 | 2.685537482 |
| MTMR14 | 0.009823939 | 0.389996363 |
| CAMK2B | 0.010691085 | 0.270453854 |
| NBPF14 | 0.003866299 | 0.410551906 |
| SLC25A15 | 0.014106711 | 3.24308747 |
| FAM118B | 0.013101773 | 2.584829197 |
| UGCG | 0.013595985 | 2.180588746 |
| CH25H | 0.001888373 | 5.377565252 |
| ZNF320 | 0.000125967 | 0.425903874 |
| CDH3 | 0.002836498 | 3.761767619 |
| TAP1 | 0.004848036 | 2.751894628 |
| FAM13A | 0.027356231 | 0.337105462 |
| PCDH1 | 0.027692649 | 0.406800526 |
| C17orf96 | 0.005854341 | 5.391761147 |
| LAMP3 | 0.003039565 | 5.784870986 |
| STRC | 0.01087953 | 0.230443464 |
| AGAP11 | 0.026502106 | 0.3285158 |
| EPHB2 | 0.010755515 | 10.50189128 |
| MOCS3 | 0.000826369 | 2.030201685 |
| VPS13D | 0.007322801 | 0.448810155 |
| C11orf73 | 0.014236711 | 2.131490082 |
| NLRP1 | 0.032278684 | 0.442002132 |
| TUBA1C | 0.000881476 | 2.222436861 |
| PRB1 | 0.032297521 | 0.370195119 |
| VARS | 0.001068572 | 2.069112967 |
| UPK2 | 0.005120208 | 22.47341982 |
| TRIM15 | 0.009160012 | 5.34825732 |
| PSAPL1 | 0.049903633 | 0.222677661 |
| LILRB3 | 0.012229087 | 4.820791748 |
| FBXL16 | 0.001946156 | 0.450524107 |
| FZD5 | 0.015078479 | 2.750416901 |
| STAT1 | 0.003978841 | 5.837817677 |
| MTHFD2 | 0.010932451 | 3.052200346 |
| ATP1B1 | 0.022827819 | 3.796812265 |
| PDSS1 | 0.005200941 | 2.244358354 |
| MCFD2 | 0.014715697 | 2.774543615 |
| NDRG4 | 0.019778301 | 2.656104156 |
| FAM43A | 0.012310942 | 3.629025233 |
| AMY2B | 0.011656165 | 0.253055168 |
| CCR1 | 0.02862406 | 7.464322436 |
| PSMC3IP | 0.002043219 | 2.025246369 |
| C5orf46 | 0.030251194 | 0.207634989 |
| MKL2 | 0.005345931 | 0.448486621 |
| TWF1 | 0.000117439 | 2.862430701 |
| TXNDC17 | 0.000476007 | 4.067961934 |
| P4HTM | 0.008726012 | 0.35897298 |
| KCNJ12 | 0.024599825 | 0.496860173 |
| SERPINB1 | 0.021674352 | 2.88850275 |
| MLKL | 0.028496532 | 4.541066101 |
| MTURN | 0.005675526 | 0.455443669 |
| BLM | 0.00080451 | 3.367610607 |
| SLC25A5 | 0.00792089 | 2.393411928 |
| C16orf59 | 0.009377468 | 2.587652829 |
| TFDP1 | 0.001511685 | 2.216589885 |
| TRIAP1 | 0.01381926 | 2.213689323 |
| IL20 | 0.016651581 | 37.11227276 |
| UQCR10 | 0.003806147 | 2.242342728 |
| C8orf89 | 0.00539372 | 0.411313449 |
| AKR1B15 | 0.009899287 | 44.32293119 |
| GPR146 | 0.021552103 | 0.435214381 |
| ABCE1 | 0.027098046 | 2.021408852 |
| ILDR1 | 0.009596027 | 0.425852706 |
| PLOD2 | 0.001922676 | 2.438071229 |
| GNAL | 0.001249567 | 0.461032644 |
| SLPI | 0.003457583 | 3.342923619 |
| NPIPB9 | 0.007308722 | 0.419665885 |
| TRIM16L | 0.002839019 | 2.349945273 |
| MST1 | 0.019290345 | 0.322381827 |
| FGD4 | 0.00474488 | 0.487519919 |
| AP2S1 | 0.001617025 | 2.863960441 |
| IL34 | 0.006310281 | 0.205396259 |
| ZFAND6 | 0.00674589 | 2.251685018 |
| FAM78A | 0.047180121 | 2.867265199 |
| RSAD2 | 0.000319189 | 14.59386503 |
| PRKCQ | 0.039258896 | 4.441383122 |
| MIB2 | 0.030911782 | 2.003326031 |
| PIK3AP1 | 0.02555037 | 3.274391026 |
| SLC5A1 | 0.012912414 | 5.14309098 |
| VN1R1 | 0.00121823 | 0.209418398 |
| PDE4DIP | 0.022778881 | 0.388148833 |
| ITGB5 | 0.011676379 | 0.461611436 |
| CXCL2 | 0.007965129 | 7.451665196 |
| PDZK1IP1 | 0.007051897 | 3.752136486 |
| ANKRD16 | 0.005745558 | 0.461902445 |
| HEG1 | 0.000901093 | 20.15706883 |
| HIST1H1B | 0.015891909 | 2.593393142 |
| SSFA2 | 0.014480787 | 0.347529843 |
| TMIGD3 | 0.046920831 | 7.857410646 |
| KLF8 | 0.016264583 | 0.46880373 |
| CCNE2 | 0.004452571 | 2.024230163 |
| CTSF | 0.046404664 | 0.45539165 |
| PRRT4 | 0.002607688 | 4.417518165 |
| SLC39A6 | 0.004674257 | 2.195392917 |
| DNAJC30 | 0.004061983 | 0.463632079 |
| APBA1 | 0.022591306 | 0.451714336 |
| RAB5C | 0.001355665 | 0.498110095 |
| GBP1 | 0.002495488 | 6.646531994 |
| FAM221A | 0.015866577 | 0.450690634 |
| HYI | 0.003498947 | 0.432426499 |
| NEUROD2 | 0.001421594 | 0.067127425 |
| FLYWCH1 | 0.039089645 | 0.217203844 |
| CPLX3 | 0.001216705 | 7.585860647 |
| HMBS | 0.011200669 | 2.360263362 |
| AIF1 | 0.00043803 | 2.365469766 |
| PRSS1 | 0.004784036 | 4.373707991 |
| CHRM1 | 0.00908748 | 0.224527695 |
| SP110 | 0.040434443 | 2.45855697 |
| IL17RE | 0.002603043 | 0.216753917 |
| GATAD2B | 0.007290914 | 0.369203055 |
| POLE2 | 0.005714924 | 3.131014824 |
| AURKB | 0.002017558 | 2.319144928 |
| JAG2 | 0.019691502 | 0.350736833 |
| CCDC96 | 0.01618314 | 2.039800063 |
| DEFB103B | 0.002538976 | 114.1909782 |
| DHRS12 | 0.005954395 | 0.472672574 |
| CHAC1 | 0.00070819 | 23.85542382 |
| RBBP6 | 0.003130249 | 2.713264695 |
| RNF225 | 0.019790469 | 3.393185037 |
| SYNE2 | 0.007994878 | 0.409234905 |
| PCSK7 | 0.026672387 | 2.606166983 |
| PPIC | 0.011267949 | 2.694882217 |
| SMPD1 | 0.008983668 | 2.811269718 |
| PAPLN | 0.020618896 | 0.234462076 |
| PLA2G2A | 0.024948608 | 16.89419697 |
| PPP4C | 0.007335068 | 2.120630817 |
| C1orf167 | 0.006493265 | 2.682070202 |
| RBM20 | 0.017470611 | 0.333089733 |
| HIST1H2AK | 0.006695261 | 4.300197765 |
| CGNL1 | 0.024053064 | 0.479690762 |
| AURKA | 0.001314603 | 3.051320484 |
| SLC4A7 | 0.004699189 | 3.74438946 |
| UBXN2A | 0.011715818 | 2.089659413 |
| CCNB2 | 0.001198178 | 4.240583082 |
| ARG2 | 0.022704224 | 4.093082891 |
| ALDH1A3 | 0.006456253 | 7.429200395 |
| CHRNA3 | 0.041984162 | 8.355957351 |
| SH3BGRL3 | 0.010283141 | 2.237449071 |
| EIF6 | 0.008769782 | 2.308016215 |
| RDH16 | 0.005501841 | 5.926183172 |
| EHF | 0.002691821 | 3.522604524 |
| IL20RB | 0.011397321 | 0.494822721 |
| DYSF | 0.020609301 | 2.268537002 |
| IL18RAP | 0.003881678 | 3.732290652 |
| PSME3 | 0.002542753 | 2.01588371 |
| SLC16A1 | 0.013187453 | 2.382220328 |
| KBTBD8 | 0.000735032 | 2.617553557 |
| LDLR | 0.001040999 | 2.48837619 |
| IL36G | 0.000753732 | 39.04989546 |
| SLC22A17 | 0.004576707 | 0.312766595 |
| NELFE | 0.02262321 | 2.742914235 |
| IL26 | 0.013270255 | 6.514606802 |
| PPP2R2C | 0.003481644 | 2.89681632 |
| ASRGL1 | 0.02250578 | 2.073857862 |
| CSF2RA | 0.006340541 | 2.548650045 |
| APOL4 | 0.032389647 | 3.854413882 |
| DLK2 | 0.045889069 | 0.354740987 |
| SPI1 | 0.000153229 | 2.052647894 |
| ZC3H12A | 0.017727271 | 4.669179601 |
| PACSIN1 | 0.009538593 | 5.661354853 |
| CCL7 | 0.001406196 | 6.783852686 |
| C10orf55 | 0.027515953 | 2.273343254 |
| LY96 | 0.030581588 | 2.503613733 |
| FAM72C | 0.005615533 | 2.652325781 |
| KIF14 | 0.000561157 | 3.113870999 |
| CCDC167 | 0.013942201 | 2.613540853 |
| CD24 | 0.004929758 | 6.438000834 |
| PLA2G3 | 0.005745482 | 5.310333399 |
| LOC643355 | 0.023680736 | 0.44946952 |
| ZNF735 | 0.00137801 | 0.430348105 |
| TTC39A | 0.000238281 | 5.510022935 |
| MRPS22 | 0.001599233 | 2.434528447 |
| HAUS7 | 0.028794538 | 0.427804801 |
| LIMD2 | 0.030102938 | 2.385368644 |
| RNF224 | 0.032858673 | 5.395694426 |
| PRSS2 | 0.008021104 | 3.602576453 |
| TRIM10 | 0.00031839 | 9.579244012 |
| GDPD1 | 0.005510759 | 0.466950313 |
| ANKRD31 | 0.00244241 | 4.932230059 |
| TYMP | 0.013532642 | 12.32850284 |
| MOB1A | 0.000843309 | 2.023733554 |
| ANKRD20A3 | 0.021342529 | 0.305105351 |
| RELT | 0.006906111 | 2.522788622 |
| TMEM105 | 0.000601666 | 3.365621462 |
| GAN | 0.004645188 | 0.299581095 |
| CACNA2D1 | 0.003513669 | 0.36467998 |
| C1QB | 0.016635929 | 7.762056999 |
| GJB6 | 0.001732036 | 5.814415715 |
| PNPLA7 | 0.008580524 | 0.130395359 |
| PGM2 | 0.001482422 | 2.956256884 |
| KCTD21 | 0.018446036 | 2.388953221 |
| SYT17 | 0.011506023 | 0.161629659 |
| SMOX | 0.002224846 | 5.456737483 |
| AES | 0.048230999 | 0.495118138 |
| AGPAT9 | 0.007739229 | 2.148288191 |
| KIFC2 | 0.004857842 | 0.313871408 |
| LFNG | 0.012961389 | 0.266644571 |
| RAPGEFL1 | 0.007512272 | 0.46332625 |
| FUT1 | 0.00047895 | 2.357388712 |
| HLA-DQB2 | 0.002089435 | 0.30383718 |
| RCOR3 | 0.008771858 | 0.471523877 |
| MT1X | 0.001419604 | 0.270258702 |
| WDR76 | 0.015013236 | 3.685112639 |
| PILRA | 0.028191483 | 2.505018986 |
| TSFM | 0.012181291 | 2.445267873 |
| HIF3A | 0.020826072 | 0.123399701 |
| ACP5 | 0.024216824 | 2.532821684 |
| USP6NL | 0.010131021 | 2.122050626 |
| AQP7 | 0.017868256 | 0.283818692 |
| CHST8 | 0.016391221 | 0.425177987 |
| TSTA3 | 0.002299156 | 2.084786164 |
| RAET1L | 0.001471126 | 6.448654997 |
| XRCC4 | 0.04336917 | 2.832031624 |
| SRSF5 | 0.009190667 | 0.471884195 |
| TLR8 | 0.007970509 | 6.466216779 |
| SLMO2 | 0.001295691 | 2.892105616 |
| RBL1 | 0.007398878 | 2.648764615 |
| LCE5A | 0.035041469 | 0.233970734 |
| HIST1H3I | 0.000626528 | 3.370302193 |
| P2RY8 | 0.045981803 | 4.256726965 |
| RASGEF1B | 0.004509798 | 0.485613459 |
| TEX30 | 0.006230431 | 4.006176608 |
| FEZF1 | 0.034214988 | 0.497752253 |
| TBXAS1 | 0.009554315 | 2.493796864 |
| FBLIM1 | 0.009524732 | 2.116195007 |
| PLEK | 0.015788201 | 4.208014547 |
| AK1 | 0.000536055 | 2.106564485 |
| TIFAB | 0.007040534 | 6.094329293 |
| TMEM45A | 0.002074785 | 2.4994662 |
| DLL1 | 0.033326376 | 0.356412132 |
| OBSL1 | 0.010323667 | 0.243755464 |
| SLC16A6 | 0.017734644 | 2.803219006 |
| AHDC1 | 0.006548534 | 0.404248965 |
| EIF1AX | 0.00277429 | 2.005384893 |
| AHNAK2 | 0.028143218 | 0.395988066 |
| BBS2 | 0.006390281 | 0.481183063 |
| SNTB1 | 0.024969122 | 0.359078009 |
| LRR1 | 0.001293355 | 2.478984051 |
| CCDC178 | 0.016337363 | 2.628981206 |
| KIF4A | 0.000142035 | 3.809414974 |
| IL4R | 0.011556881 | 2.882812978 |
| ACTR3 | 0.003534952 | 2.461220954 |
| FAM65C | 0.002541758 | 3.027111235 |
| SLC46A2 | 0.035379343 | 0.348222437 |
| MACROD2 | 0.00094758 | 0.282032093 |
| ERCC6L | 0.00082861 | 4.368891749 |
| AFAP1L2 | 0.013429762 | 2.047430641 |
| ZNF675 | 0.001452056 | 0.376700799 |
| TRIM45 | 0.003032025 | 0.381770733 |
| EIF4EBP1 | 0.00146973 | 2.643760181 |
| CROCC | 0.033690765 | 0.463665999 |
| STK19 | 0.005615182 | 3.731662861 |
| LRIG1 | 0.020553714 | 0.499790184 |
| E2F3 | 0.010887078 | 2.102519654 |
| NEURL3 | 0.047876226 | 6.591053545 |
| UBE2C | 0.020387498 | 2.14338075 |
| NRBF2 | 0.013565076 | 2.438059478 |
| TMEM154 | 0.005322802 | 2.477191745 |
| PFDN6 | 0.00928154 | 2.128100498 |
| C5orf45 | 0.027679058 | 0.423028757 |
| CARD6 | 0.001935997 | 6.192353659 |
| SOX7 | 0.043971479 | 2.704424839 |
| SLC22A15 | 0.029204778 | 0.364702072 |
| TTC38 | 0.005349507 | 0.361936356 |
| CCND1 | 0.00120652 | 0.419852207 |
| FBXO45 | 0.001028805 | 2.070165404 |
| LCE3D | 0.002315399 | 39.08161567 |
| TMEM167A | 0.008617423 | 2.129697085 |
| RORA | 0.009899472 | 0.450097672 |
| NRF1 | 0.020328604 | 0.454856368 |
| ADRB1 | 0.03713305 | 0.437943519 |
| CLEC2A | 0.003281217 | 0.32305446 |
| PIK3R1 | 0.010675775 | 0.35559971 |
| ENDOV | 0.001914663 | 0.361915437 |
| FUOM | 0.000681529 | 2.960626097 |
| TMEM63C | 0.046995382 | 0.148800829 |
| CCNA2 | 0.002757213 | 2.799554138 |
| BFSP1 | 0.039877939 | 2.319717277 |
| MSMP | 0.006983106 | 2.921970203 |
| DUS2 | 0.000196855 | 2.383840055 |
| GGH | 0.005324671 | 3.585590884 |
| TTC24 | 0.008341685 | 2.243800365 |
| PRODH | 0.016458518 | 0.312336192 |
| IRF9 | 0.000523654 | 2.442579422 |
| SCD5 | 0.02999751 | 0.293171329 |
| ZNF43 | 0.032111159 | 0.434984672 |
| TOP2A | 0.005918943 | 2.523147074 |
| GSDMA | 0.024468303 | 2.703333475 |
| BDKRB2 | 0.000373065 | 2.03817126 |
| GART | 0.000301501 | 2.203016336 |
| ADAMDEC1 | 0.012991242 | 81.82542551 |
| DUSP9 | 0.014710952 | 4.279393197 |
| CCNE1 | 0.019331779 | 4.754948453 |
| ODC1 | 0.009575594 | 2.679729991 |
| PSMB5 | 0.000658577 | 2.146559912 |
| SHD | 0.000223136 | 2.03903818 |
| LAT2 | 0.023655498 | 2.668958524 |
| PITX1 | 0.000888073 | 19.75919235 |
| WNT2B | 0.007553196 | 0.497888232 |
| RGS20 | 0.00462972 | 5.348396517 |
| GALE | 0.010753358 | 2.65857861 |
| ZNF439 | 0.002048921 | 0.380698476 |
| PPP4R1 | 0.000730192 | 2.302146618 |
| HIGD1A | 0.000119004 | 3.769769955 |
| KANK3 | 0.016436297 | 0.485921634 |
| GREM2 | 0.015122803 | 0.459527631 |
| DYM | 0.001379431 | 2.169770253 |
| CYB5R4 | 0.004766723 | 3.277870985 |
| SQRDL | 0.006331404 | 2.19266611 |
| C15orf56 | 0.048436692 | 2.15077806 |
| MRPL12 | 0.004006644 | 2.708893222 |
| POC1A | 0.003832725 | 3.28749878 |
| MAST1 | 0.026312952 | 0.426639287 |
| INA | 0.012464041 | 5.093340654 |
| OXCT1 | 0.008848143 | 2.098544204 |
| TGM3 | 0.001701165 | 6.04334635 |
| FCGR1A | 0.003293988 | 64.66694882 |
| GPRIN1 | 0.036643012 | 3.439169692 |
| PANX1 | 0.000920302 | 2.625682724 |
| BSDC1 | 0.025347612 | 0.465912394 |
| TGM2 | 0.015398813 | 4.464890632 |
| OVCH2 | 0.037983346 | 0.285170442 |
| CNFN | 0.000239013 | 7.04668794 |
| FCMR | 0.007107926 | 3.140865588 |
| EXD3 | 0.008335247 | 0.337762821 |
| FAM111B | 0.046173749 | 2.581011815 |
| CATSPERB | 0.010763295 | 3.353851393 |
| P2RX6 | 0.002553219 | 0.359830017 |
| CSTA | 0.000398629 | 5.47949593 |
| FKBP10 | 0.017143316 | 0.410322315 |
| FZD2 | 0.043316801 | 2.531180745 |
| TNNC1 | 0.041853167 | 0.314313888 |
| ECT2 | 9.25829E-05 | 3.917421098 |
| IRAK2 | 0.01086475 | 5.171394911 |
| HS3ST1 | 0.027593889 | 2.84719361 |
| RNF213 | 0.022343982 | 3.367274054 |
| TMEM117 | 0.010336693 | 2.153053399 |
| PRICKLE2 | 0.029262492 | 2.289132095 |
| FAM213B | 0.027547286 | 2.296431398 |
| DNMBP | 0.006198902 | 0.441180622 |
| ECHDC3 | 0.019022644 | 0.492078068 |
| NTN5 | 0.021751898 | 0.267092031 |
| SNRPD1 | 0.003782513 | 2.391983111 |
| KIF20B | 0.001220123 | 2.228488873 |
| SPIN4 | 0.001042427 | 4.185377661 |
| SKA3 | 0.002022733 | 4.959230192 |
| CNTFR | 0.00423827 | 0.141029176 |
| QPCT | 0.019786798 | 2.361301269 |
| SEC61G | 0.000753125 | 2.208519651 |
| FRMD8 | 0.001719849 | 2.438004571 |
| LYAR | 0.008343103 | 2.659908677 |
| MSR1 | 0.040812178 | 6.354233885 |
| UNC93A | 0.003291007 | 5.237780073 |
| CMYA5 | 0.002473824 | 0.257444051 |
| IL17RC | 0.000368603 | 0.491910923 |
| IFI6 | 0.000358305 | 12.31418617 |
| HDAC5 | 0.011331203 | 0.382349231 |
| RNF25 | 0.009022899 | 2.068161094 |
| EIF4A3 | 0.005402838 | 2.491107812 |
| ARHGEF28 | 0.006507624 | 0.35830775 |
| NCF2 | 0.001154411 | 2.687386753 |
| C3AR1 | 0.04574258 | 4.366229241 |
| ITPKB | 0.025309047 | 0.491469729 |
| TNFAIP2 | 0.029313108 | 2.05973859 |
| CRCT1 | 0.012403337 | 2.955984587 |
| PEX6 | 0.000551037 | 0.460020331 |
| ADGRE1 | 0.026860298 | 3.456968871 |
| CNGA1 | 0.005686283 | 0.268090924 |
| SYNGR1 | 0.00684929 | 0.411706716 |
| ZNF84 | 0.009509351 | 0.390011574 |
| DYNLT1 | 0.012807619 | 2.455795524 |
| LCE3B | 0.000123478 | 96.99642706 |
| LGMN | 0.048033015 | 2.479732243 |
| CABP2 | 0.003131567 | 24.12836224 |
| B4GALNT4 | 0.026230197 | 0.443086274 |
| GOLGA8N | 0.015974515 | 0.429643469 |
| ZWILCH | 0.000989437 | 2.121752603 |
| SOX4 | 0.031101549 | 0.482661737 |
| SAMD9 | 0.005565997 | 9.308375956 |
| HIST1H2BI | 0.001474582 | 3.126735881 |
| SGOL1 | 0.00152186 | 2.884612499 |
| TMPRSS4 | 0.020490719 | 6.260311595 |
| ZNF540 | 0.011054527 | 0.354485239 |
| B4GAT1 | 0.011548038 | 0.475022294 |
| KIF26A | 0.004125937 | 0.356685146 |
| FSCN1 | 0.003135358 | 3.506675703 |
| FCN1 | 0.015867466 | 13.41283432 |
| SLC48A1 | 0.013814232 | 0.495848107 |
| TFEC | 0.028264518 | 4.39731916 |
| SLC26A4 | 0.021081261 | 10.58587946 |
| HMOX1 | 0.006115081 | 3.352879766 |
| MT1A | 0.031076913 | 2.502929701 |
| KY | 0.021636057 | 0.362412183 |
| SH3GL3 | 0.028853807 | 2.94343391 |
| ERVMER34-1 | 0.025359291 | 2.566792752 |
| SPATA24 | 0.021349347 | 2.61125097 |
| RAC3 | 0.001278575 | 0.239408095 |
| CMPK2 | 0.000711688 | 13.08921036 |
| GPR180 | 0.027061156 | 24.51762765 |
| PRR11 | 0.000302652 | 3.217276098 |
| HYAL4 | 0.027050286 | 12.15866351 |
| SELE | 0.017004621 | 2.245583661 |
| XDH | 0.032324129 | 6.106585646 |
| CDK1 | 0.002616438 | 2.661899201 |
| ALPL | 0.00936802 | 2.835673801 |
| PLA2G4E | 0.007505949 | 3.026921117 |
| THEMIS2 | 0.033472706 | 3.516784448 |
| CERS3 | 0.024853668 | 2.417762794 |
| TGFA | 0.03681895 | 2.578046778 |
| DSG3 | 0.002255196 | 3.726834628 |
| HRSP12 | 0.002851359 | 2.432055367 |
| SCIN | 0.021384489 | 0.306038819 |
| CHP2 | 0.004411734 | 0.312403969 |
| PDCD2L | 0.017056606 | 2.404849195 |
| FOXRED2 | 0.00285933 | 2.755155717 |
| SPRR2D | 0.011173047 | 28.17762558 |
| SLC36A1 | 0.011903233 | 2.025516624 |
| SLC18A2 | 0.039348238 | 0.326971294 |
| NME1 | 0.010310318 | 2.156452635 |
| GBP3 | 0.006474454 | 3.56616236 |
| PTP4A1 | 0.026395116 | 2.170304799 |
| KATNB1 | 0.006457828 | 2.013766646 |
| ADH1A | 0.001635524 | 0.382943294 |
| B3GAT3 | 0.007507417 | 0.461973574 |
| CLEC4C | 0.011671476 | 5.289606896 |
| KIF20A | 0.000510143 | 3.878157624 |
| PLCD4 | 0.001932921 | 4.42708447 |
| HS3ST6 | 0.003495427 | 0.209865514 |
| HES6 | 0.042761043 | 2.18633336 |
| GMPPB | 0.002136867 | 3.23899152 |
| RHBDL1 | 0.006693132 | 0.358360983 |
| REN | 0.000763267 | 23.18250828 |
| SPRR1A | 0.019057074 | 35.15877905 |
| DEPDC1B | 0.001772433 | 3.716976248 |
| RHOXF1 | 0.018598762 | 0.398707714 |
| CXCL11 | 0.033656231 | 8.079880112 |
| LONRF1 | 0.000625762 | 0.323152351 |
| ABI3 | 0.003734698 | 2.060775735 |
| PIFO | 0.02005831 | 0.285308749 |
| CNOT6 | 0.000603204 | 2.078529669 |
| CFAP45 | 0.004100841 | 2.119220669 |
| ASB16 | 0.000840975 | 2.090101956 |
| SLC8A2 | 0.001972195 | 3.169912301 |
| TPBG | 0.000293734 | 2.865917872 |
| KPNA5 | 0.006395849 | 2.194033339 |
| PLXNA3 | 0.006363637 | 0.341293652 |
| HIST1H4I | 0.00321226 | 4.678543084 |
| HS3ST3B1 | 0.003050302 | 2.403921267 |
| SORCS3 | 0.034998155 | 0.482814856 |
| GZMM | 0.03110143 | 2.622902384 |
| NUDT15 | 0.002941812 | 2.910670821 |
| HGH1 | 0.001225733 | 2.930498033 |
| EFCAB7 | 0.005897054 | 0.474912096 |
| ADH1C | 0.002716935 | 0.391492832 |
| H2AFX | 0.026422281 | 2.114405996 |
| TRIM22 | 0.004919606 | 4.904289498 |
| RASGRP1 | 0.01030669 | 2.299711855 |
| SRFBP1 | 0.006594675 | 2.208211802 |
| AXIN2 | 0.024782526 | 0.456050752 |
| OASL | 2.98471E-05 | 18.71579399 |
| ANO9 | 0.04668749 | 0.40934851 |
| IL4I1 | 0.013068717 | 10.45732158 |
| DTX3L | 0.006937079 | 3.142914246 |
| NAMPT | 0.018241673 | 3.739546134 |
| LINGO4 | 0.021873149 | 0.255948276 |
| COL22A1 | 0.041970551 | 2.569191488 |
| HEPHL1 | 0.002301523 | 22.79190047 |
| CACNA2D2 | 0.011958248 | 0.323858576 |
| NT5C3A | 0.006451594 | 3.285893712 |
| MEI1 | 0.040736789 | 2.424651665 |
| APOE | 0.001183213 | 0.331124314 |
| ZPR1 | 0.003773462 | 2.017481861 |
| SLC1A1 | 0.036227501 | 2.259509475 |
| IFIH1 | 0.004571179 | 4.664531753 |
| TACC3 | 0.015742264 | 2.85527647 |
| RND1 | 1.61618E-05 | 13.08867925 |
| PXDN | 0.035911285 | 2.147541256 |
| AAMDC | 0.002996424 | 0.412197145 |
| PAIP2B | 4.98051E-05 | 0.32280869 |
| GS1-259H13.2 | 0.017467253 | 0.385196641 |
| SPIB | 0.002480651 | 10.00755251 |
| IFI44 | 0.002147064 | 6.846114905 |
| ARL5B | 0.00954313 | 2.352258586 |
| KAT2A | 0.007895285 | 0.42622324 |
| ELOVL4 | 0.007218723 | 2.450697496 |
| C1orf105 | 0.030430898 | 2.988990779 |
| PLXNB1 | 0.010990796 | 0.468637979 |
| RALA | 0.000330736 | 2.748816432 |
| STRN3 | 0.000604137 | 2.008602444 |
| TRPV4 | 0.008496047 | 0.489687535 |
| PTGR1 | 0.014204739 | 2.203650809 |
| FAM110C | 0.013557216 | 4.318738796 |
| CASP12 | 0.016365108 | 0.477345906 |
| USMG5 | 0.002028105 | 2.835901447 |
| CDCA2 | 0.007194618 | 3.206700514 |
| CHAC2 | 0.000792577 | 4.948986754 |
| EPN2 | 0.004811487 | 0.490162819 |
| DZIP1L | 0.024869672 | 0.295914358 |
| UBE2L6 | 0.001310066 | 4.59412152 |
| RAD51 | 0.005317286 | 5.818989986 |
| PRSS27 | 0.000413257 | 22.02355603 |
| LOC728392 | 0.006404628 | 0.327585873 |
| ZNF429 | 0.049629982 | 0.363538099 |
| OR2W3 | 0.03867379 | 2.395751997 |
| CLDN8 | 0.013667207 | 0.462325186 |
| TMEM116 | 0.00086421 | 0.331483585 |
| SLC24A4 | 0.011669886 | 2.376888932 |
| LYPD1 | 0.0064016 | 5.107159383 |
| TMEM255A | 0.014539648 | 0.355990005 |
| DCANP1 | 0.011157445 | 3.001874452 |
| NTRK2 | 0.022535908 | 0.378013781 |
| PLK1 | 0.004262096 | 2.928171743 |
| STEAP1B | 0.031438574 | 3.352857337 |
| HIST1H1D | 0.007102126 | 3.841041111 |
| TNFRSF11A | 0.032348848 | 2.280244789 |
| SPRR2F | 0.01593032 | 18.12580285 |
| SIN3B | 0.016613693 | 0.492085715 |
| SCNN1D | 0.004981751 | 2.029034558 |
| C16orf95 | 0.00455141 | 2.096654437 |
| CDCA5 | 0.012444984 | 2.805347768 |
| SYNE1 | 0.00870342 | 0.429456334 |
| FABP5 | 0.001859277 | 13.07201807 |
| PSME2 | 0.001002038 | 2.771429229 |
| GBP2 | 0.025526192 | 2.215285534 |
| ZNF479 | 0.005349204 | 0.413866353 |
| CAT | 0.002768443 | 0.474667802 |
| C10orf99 | 0.000598088 | 70.43217678 |
| BUB1B | 0.002708169 | 2.466998242 |
| LCT | 0.042824751 | 2.59582421 |
| WARS | 0.003793953 | 4.137850915 |
| LAMB1 | 0.003572268 | 0.459442049 |
| BIRC5 | 0.044599934 | 2.614455326 |
| SOX12 | 0.014136581 | 0.425985733 |
| DDX60 | 0.002699684 | 8.387515804 |
| NEK2 | 0.000348053 | 3.052134282 |
| LLPH | 0.000572637 | 2.070552406 |
| LCN2 | 0.005743111 | 84.8171515 |
| TRUB2 | 0.006118966 | 2.04523339 |
| GGCT | 0.007028355 | 3.686750113 |
| FAM166B | 0.001364562 | 0.061730727 |
| ATF6B | 0.005201236 | 0.411084226 |
| FCGR3B | 0.02470641 | 17.40572688 |
| TCN1 | 0.00478574 | 26.36609214 |
| MPO | 0.002928445 | 4.814730538 |
| CHCHD3 | 0.001836498 | 2.137866318 |
| ARMT1 | 0.007069458 | 2.105130972 |
| CSF3R | 0.00094865 | 3.896049707 |
| PARK2 | 0.016947124 | 0.490597532 |
| PGC | 0.032482582 | 0.281556682 |
| DHRS7 | 0.000586499 | 2.84682798 |
| WIF1 | 0.003722525 | 0.030981334 |
| AEN | 0.000865186 | 3.102690616 |
| SLC26A9 | 0.009580945 | 6.458337356 |
| RYR1 | 0.001198878 | 0.462910565 |
| AGAP9 | 0.011660653 | 0.481124192 |
| CFAP70 | 0.003602731 | 0.326083678 |
| ASCC3 | 0.002524049 | 2.262911981 |
| ATP6V0D1 | 0.005169035 | 2.219207524 |
| SLC2A6 | 0.032386754 | 3.19993885 |
| CCDC85C | 0.015499234 | 2.690922394 |
| TMOD1 | 0.001234533 | 19.77006836 |
| ZC3H7B | 0.006037615 | 0.378769098 |
| TNFSF15 | 0.00364633 | 3.062537786 |
| UBXN8 | 0.010066768 | 2.239194841 |
| MCTS1 | 0.000310432 | 2.610513275 |
| DISC1 | 0.011872019 | 2.130943752 |
| CNN2 | 0.000390041 | 2.121861211 |
| ZBED2 | 0.020999439 | 4.318203426 |
| RPL27A | 0.028571294 | 2.52065698 |
| C19orf18 | 0.009054713 | 0.353661825 |
| NEIL1 | 0.031405176 | 0.359274994 |
| LDHA | 0.004215571 | 2.355726989 |
| KRT77 | 0.007838183 | 0.061457297 |
| ANKRD2 | 0.030976477 | 0.273387103 |
| HES4 | 0.000273358 | 0.360014783 |
| ADHFE1 | 0.032577956 | 0.441224039 |
| NR3C2 | 0.033333428 | 0.466196742 |
| RAB31 | 0.039972232 | 3.050844245 |
| SIGLEC14 | 0.024950064 | 6.496446695 |
| CDK5R1 | 0.012262195 | 3.337626014 |
| TNFAIP6 | 0.01495444 | 4.597089048 |
| GBP5 | 0.003354159 | 8.817017415 |
| GPRIN3 | 0.023414999 | 3.262204184 |
| GJB2 | 0.008918413 | 23.1199186 |
| KRT16 | 0.015483513 | 15.75732203 |
| NUSAP1 | 0.001317184 | 2.181981102 |
| WDFY4 | 0.049425212 | 3.192629242 |
| MANF | 0.004732942 | 2.208497644 |
| ARC | 0.00229547 | 3.397754945 |
| SEC23B | 0.00948457 | 2.053703538 |
| MGAT2 | 0.002030431 | 2.057473456 |
| GOLGA8A | 0.01017871 | 0.488926806 |
| DEFB4A | 0.000160473 | 3505.251346 |
| KIF23 | 0.000584298 | 2.742721862 |
| HIST1H3G | 0.002352668 | 3.924977349 |
| LCE3C | 0.023883108 | 31.56687879 |
| PKMYT1 | 0.0066958 | 3.17549952 |
| ADGRB1 | 0.022618941 | 0.11801688 |
| HDHD1 | 0.000103329 | 3.364128887 |
| NAB1 | 0.026864126 | 0.453710629 |
| PDK4 | 0.039177878 | 0.333720832 |
| MAZ | 0.01138428 | 2.08037555 |
| GSG2 | 0.036728503 | 3.200097435 |
| ELF3 | 0.012043035 | 4.657391425 |
| IRF1 | 0.004630265 | 7.693177657 |
| MTHFD1L | 0.016545077 | 2.193727123 |
| AGAP6 | 0.011835142 | 0.499089397 |
| GJB4 | 0.037223915 | 0.289881436 |
| MRPL32 | 0.000755244 | 2.817698692 |
| SLC25A48 | 0.049242998 | 0.355612988 |
| BTK | 0.011261078 | 3.787030084 |
| SLC4A11 | 0.031588468 | 2.246762476 |
| IQGAP3 | 0.016761755 | 2.174221513 |
| ABHD11 | 0.015178751 | 0.447551273 |
| GATM | 0.000329315 | 0.256654997 |
| FAM69B | 0.014880997 | 0.442358126 |
| GTPBP4 | 0.02651396 | 2.132586641 |
| SOX5 | 3.36891E-05 | 0.418231335 |
| C12orf56 | 0.009098155 | 5.998340147 |
| VNN1 | 0.042045457 | 4.284732715 |
| EDAR | 0.001280279 | 0.44229636 |
| SPINK1 | 0.038351944 | 0.069324218 |
| IL19 | 0.032962004 | 148.1223173 |
| KIF11 | 0.000786362 | 2.149862887 |
| IL12B | 0.005989137 | 11.35527326 |
| ARL2BP | 0.001265618 | 2.160212631 |
| WSCD1 | 4.81307E-05 | 0.146976575 |
| ABHD17C | 0.008766214 | 2.392906516 |
| GJA5 | 0.026236471 | 3.377132255 |
| IFI30 | 0.01276454 | 2.989193245 |
| MRPS17 | 0.008739613 | 2.274192787 |
| ZNF490 | 0.004451174 | 0.478071468 |
| ZNF793 | 0.018717408 | 0.458730948 |
| BCL10 | 0.013894474 | 2.548240707 |
| RARRES3 | 0.049197966 | 2.361367824 |
| ASF1B | 0.031656126 | 2.437636867 |
| ASPG | 0.02949413 | 3.381373348 |
| VMP1 | 0.013602212 | 2.461921779 |
| PPARD | 0.037782325 | 2.772548504 |
| CSF2RB | 0.012661482 | 2.552167834 |
| DCST2 | 0.012945156 | 0.235128716 |
| NPIPA2 | 0.021848321 | 0.41966373 |
| CKB | 0.014712911 | 0.396017436 |
| HIF1A | 0.009065775 | 2.004095954 |
| TPSG1 | 0.049902599 | 0.345600409 |
| BATF2 | 0.000237334 | 16.87465997 |
| CLLU1OS | 0.001094853 | 6.93597303 |
| C11orf52 | 0.005022246 | 0.438813879 |
| PNP | 0.00134109 | 4.392213048 |
| IL17RD | 0.021366589 | 0.485105807 |
| RMI2 | 0.000378883 | 2.198245139 |
| RAD54L | 0.011050158 | 2.330814075 |
| SLC7A11 | 0.005621677 | 5.85468188 |
| UCN | 0.0091681 | 0.444782529 |
| CENPE | 0.000358267 | 2.49343966 |
| UTP11L | 0.000870721 | 2.131031999 |
| MFRP | 0.03804702 | 2.526756957 |
| MRPL47 | 0.001860498 | 2.122861234 |
| GSE1 | 0.000980222 | 0.437924595 |
| MPHOSPH6 | 0.005031707 | 3.66100668 |
| ADAM12 | 0.037180209 | 3.66917525 |
| TNFRSF25 | 0.042205073 | 0.390216469 |
| S100A2 | 0.003472535 | 4.65901296 |
| LCK | 0.043206095 | 3.556048562 |
| TNFRSF10A | 0.016708443 | 2.093960552 |
| DUX4 | 0.009810457 | 0.421056105 |
| SERPINB9 | 0.000942894 | 6.468627345 |
| WNT5A | 0.019973824 | 5.801468732 |
| CTSL | 0.003422058 | 3.634121466 |
| ARPC3 | 0.005970933 | 2.180713632 |
| DDX28 | 0.003885925 | 2.077288629 |
| WNT3A | 0.045384896 | 0.436925087 |
| MPZL2 | 0.001960122 | 7.464765461 |
| PHYHD1 | 0.00848601 | 0.240470359 |
| PGAM5 | 0.010185132 | 2.817219802 |
| IL11RA | 0.016261557 | 0.379272175 |
| SLC26A11 | 0.02166274 | 0.329280492 |
| SOCS1 | 0.026924433 | 3.499197606 |
| TMEM86A | 0.009792282 | 4.160693796 |
| TCF7 | 0.029355744 | 2.152829171 |
| ARPC4-TTLL3 | 0.032689541 | 0.492877832 |
| ID4 | 0.01397882 | 0.263261363 |
| NDUFAF4 | 0.013297894 | 2.616841129 |
| PLA2G6 | 0.000232982 | 0.496806159 |
| EFCAB1 | 0.049372502 | 0.452633502 |
| C1QL4 | 0.037410233 | 0.263951516 |
| RELB | 0.040822768 | 2.143656612 |
| GLRX2 | 0.032733299 | 2.133716364 |
| KIF2C | 0.005330485 | 2.82024616 |
| ALDH8A1 | 0.000836181 | 0.398780735 |
| SERPINB4 | 0.009669961 | 780.4238703 |
| PTPN21 | 0.04133661 | 0.419166316 |
| IFRD2 | 0.012619434 | 3.72134262 |
| ENTPD7 | 0.010830902 | 2.513209652 |
| OSR2 | 0.02372497 | 0.454707077 |
| ACE2 | 0.001887256 | 3.79166583 |
| ZBP1 | 0.033861934 | 8.842306739 |
| ZNF652 | 0.013721517 | 0.451612343 |
| FCGR2C | 0.008623503 | 3.00718655 |
| PARP9 | 0.001057992 | 4.247669504 |
| ZBTB8OS | 0.00316542 | 2.170084999 |
| TVP23C-CDRT4 | 0.00670364 | 0.488003524 |
| IQSEC2 | 0.021568536 | 0.460105895 |
| SYT9 | 0.025223371 | 0.082959796 |
| CDHR1 | 0.021854688 | 0.243085728 |
| PTAFR | 0.016240651 | 4.487725487 |
| ABCC3 | 0.010915093 | 0.408773135 |
| PLBD1 | 0.00188256 | 4.478736023 |
| PKD1 | 0.027643904 | 0.463150973 |
| ARHGEF10 | 0.045787493 | 0.451086376 |
| GTSF1 | 0.013251029 | 5.041395296 |
| KIFC1 | 0.000677998 | 2.564146293 |
| GPC1 | 0.000603822 | 0.417266132 |
| MSX2 | 0.007712746 | 2.0226661 |
| LRRC20 | 0.000449998 | 2.876884971 |
| TNS2 | 0.045313165 | 0.395197562 |
| OAS2 | 0.001365175 | 10.13714036 |
| HAPLN3 | 0.010608358 | 3.000474854 |
| ZAN | 0.026465866 | 3.897957967 |
| FAM83D | 0.020948606 | 2.085286256 |
| ACOT11 | 0.009474937 | 2.940051186 |
| PSMB10 | 0.021912613 | 2.084402462 |
| POLB | 0.001864772 | 2.521839756 |
| EAF2 | 0.005313656 | 2.909971287 |
| RTP4 | 0.01307806 | 6.847565813 |
| SLC25A10 | 0.025594437 | 2.820738023 |
| AKR1B10 | 0.000304381 | 125.7845403 |
| PLEKHN1 | 0.018763574 | 0.273031837 |
| ACRC | 0.005491347 | 2.107338758 |
| VWA2 | 0.016546823 | 0.383778428 |
| UBE3D | 0.024682795 | 2.133529003 |
| PLAUR | 0.008477066 | 2.361934747 |
| HAL | 0.007402075 | 3.604812211 |
| RND3 | 0.031894878 | 2.043138639 |
| PAPL | 0.000175726 | 8.920575083 |
| BUB1 | 0.041748167 | 3.020075014 |
| SLC26A5 | 0.001091779 | 0.151421136 |
| CSAD | 0.008431799 | 0.441021951 |
| PAK1IP1 | 0.00188743 | 2.427797165 |
| CXCL9 | 0.018770907 | 66.573381 |
| PLCB3 | 0.018883267 | 4.142515375 |
| LCE3A | 0.001023951 | 152.0012187 |
| CACNG8 | 0.023640263 | 0.344917565 |
| TRPM6 | 0.019929414 | 2.442748316 |
| PTTG2 | 0.023817078 | 2.644543657 |
| CCDC68 | 0.015610201 | 0.497658055 |
| COTL1 | 0.035085664 | 3.048714284 |
| UPK3A | 0.045661345 | 2.255709025 |
| TRIM69 | 0.005085177 | 2.20089655 |
| PSMA6 | 0.003757143 | 2.052836772 |
| LAD1 | 0.006877574 | 2.479927964 |
| EPHX3 | 0.006825401 | 2.379414058 |
| HEATR5A | 0.03207697 | 2.121910736 |
| CBX2 | 0.01381838 | 3.174951375 |
| BTBD16 | 0.008307602 | 0.430932738 |
| NPIPA8 | 0.025346998 | 0.410414922 |
| PTPRU | 0.026664601 | 0.410353502 |
| UBE2NL | 0.001665864 | 3.107119431 |
| ETV3L | 0.039750233 | 2.609114817 |
| IL23A | 0.042236408 | 4.956284808 |
| FBXO5 | 0.004899594 | 2.065242015 |
| XKR6 | 0.037929262 | 0.498637965 |
| COL23A1 | 0.004549315 | 0.301397582 |
| FCGR1B | 0.025559523 | 3.691810249 |
| TNFRSF18 | 0.031894665 | 0.387014571 |
| IL6 | 0.003947722 | 4.136593663 |
| SGK1 | 0.00446799 | 3.200865866 |
| PPP1R10 | 0.000212453 | 0.374129516 |
| FGFBP1 | 0.030849882 | 4.266851899 |
| PSMD12 | 0.012961268 | 2.244156365 |
| CYP4F22 | 0.029772275 | 2.009947364 |
| PGLYRP2 | 0.001010471 | 28.19467496 |
| BIRC3 | 0.02968624 | 4.049862933 |
| HPDL | 0.003335785 | 5.429231799 |
| GABRD | 0.012194522 | 0.334792812 |
| SRGAP2 | 0.00707283 | 0.46686982 |
| KCNK10 | 0.017363724 | 2.439072614 |
| MTCL1 | 0.020773445 | 3.189720282 |
| PTPN14 | 0.016987845 | 0.460233662 |
| CTLA4 | 0.028968037 | 4.364011636 |
| LTB4R2 | 0.002771309 | 2.58874782 |
| AP1S3 | 0.009540177 | 3.405147268 |
| NAGS | 0.002351118 | 2.141174306 |
| MECOM | 0.002346308 | 2.635903928 |
| HIST1H2BH | 0.017259447 | 3.031487446 |
| SH2D5 | 0.020080051 | 7.03411586 |
| ICOS | 0.039245398 | 7.321407197 |
| PPP1R32 | 0.025870658 | 0.49206585 |
| PARP14 | 0.002393756 | 2.866305403 |
| LYN | 0.014905628 | 4.375372091 |
| EPHX2 | 0.011289311 | 0.402982299 |
| LRRC8B | 0.006158521 | 3.354939809 |
| ADCK3 | 0.009672374 | 0.393155368 |
| CEACAM19 | 0.011513957 | 3.162453668 |
| ELFN2 | 0.004310643 | 0.238553961 |
| SYS1 | 0.004137578 | 2.145195977 |
| MTFR2 | 0.002010433 | 2.027539043 |
| GZMB | 0.00157458 | 35.80931898 |
| ETHE1 | 0.02774158 | 2.849340859 |
| ATOX1 | 0.001564384 | 2.259794368 |
| FAM64A | 0.012402717 | 2.069955345 |
| TNFSF12-TNFSF13 | 0.047505003 | 2.338879747 |
| IL36A | 0.002761058 | 149.663925 |
| KCNS3 | 0.02711168 | 2.295846859 |
| UBE2F | 0.006662232 | 3.069884233 |
| UBE2T | 0.007488392 | 2.423604468 |
| ALG3 | 0.001145398 | 2.212505315 |
| EIF2B2 | 0.017757956 | 2.248734831 |
| HNRNPCL4 | 0.002483163 | 2.157739384 |
| GNL2 | 0.018407208 | 2.007760765 |
| ZNF703 | 0.003579505 | 0.429536513 |
| DCUN1D3 | 0.003528695 | 2.930948533 |
| CXCR2 | 0.003523083 | 8.27682082 |
| ADGRF2 | 0.015613025 | 0.287903591 |
| ADAMTS4 | 0.00378447 | 15.31244028 |
| IRF8 | 0.007015905 | 4.834783079 |
| ADGRG5 | 0.024566144 | 2.413215748 |
| CLASRP | 0.010146112 | 0.369103821 |
| C15orf48 | 0.00128859 | 3.783604966 |
| OAF | 0.044531959 | 2.333704679 |
| HSPA4 | 0.000255133 | 2.033937841 |
| MX2 | 0.005422859 | 4.693836325 |
| CNTN2 | 0.023597388 | 0.24122754 |
| GLTPD2 | 0.021370805 | 5.953944864 |
| MID1IP1 | 0.003370865 | 2.541097752 |
| MAOA | 0.031891923 | 0.418713748 |
| ACPP | 0.005075187 | 2.575855007 |
| ZSCAN18 | 0.002851982 | 0.284232167 |
| CCDC124 | 0.0014302 | 2.000395832 |
| GBP6 | 0.040632639 | 5.013197953 |
| GFOD2 | 0.004494067 | 2.891585919 |
| CCDC114 | 0.008160245 | 0.093676378 |
| ZFP14 | 0.035180557 | 0.381513144 |
| UVSSA | 0.029386106 | 0.487281532 |
| E2F7 | 0.001567283 | 2.447405254 |
| MEGF6 | 0.021413679 | 0.392374827 |
| BAIAP2L2 | 0.011158211 | 2.093072579 |
| ZNF862 | 0.010580753 | 0.491113502 |
| SLC47A1 | 0.013314775 | 0.384192328 |
| TACC2 | 0.005463003 | 0.398266767 |
| HS3ST3A1 | 0.006485767 | 2.296642394 |
| FCRLB | 0.039591125 | 4.252878288 |
| C1orf53 | 0.03456894 | 2.586734315 |
| IL1RN | 0.022017446 | 2.086741393 |
| FAM117A | 0.016051282 | 0.372662071 |
| CHRNA9 | 0.020724619 | 15.47004144 |
| FAM229A | 0.00723707 | 0.342202068 |
| ZNF559 | 0.00755362 | 0.49491037 |
| ELMSAN1 | 0.007766509 | 0.432082175 |
| SEPSECS | 0.000763647 | 0.489837965 |
| TTC9 | 0.005596588 | 3.439569851 |
| CATSPERG | 0.004709236 | 0.149291951 |
| FAIM | 0.020743359 | 2.106549244 |
| PYCR1 | 0.008174062 | 2.625059368 |
| METTL6 | 0.003419025 | 2.112465673 |
| HMGB3 | 0.04002225 | 2.05956695 |
| RHCG | 0.003345082 | 79.02118603 |
| MX1 | 0.000653686 | 10.38367416 |
| ZNF726 | 0.022695353 | 0.401657964 |
| SAMD9L | 0.042628489 | 3.378763846 |
| TREX2 | 0.001221391 | 6.293318658 |
| SLC7A7 | 0.025945541 | 2.919844749 |
| CXCL10 | 0.009436208 | 79.80150422 |
| NRN1L | 0.007897948 | 0.410039671 |
| GDPD3 | 0.008395958 | 3.444471696 |
| PGBD5 | 0.033505049 | 6.231946993 |
| NUF2 | 0.000473857 | 2.031492179 |
| FOS | 0.008458063 | 0.11753303 |
| CASP10 | 0.014623109 | 2.0277895 |
| LTB | 0.027254228 | 4.765821943 |
| ATG16L2 | 0.011101366 | 0.450288709 |
| ZNF415 | 0.014613835 | 0.292658554 |
| HOOK2 | 0.003842625 | 0.476338851 |
| SLAMF7 | 0.000981738 | 8.096065452 |
| SLC5A10 | 0.034235582 | 2.033942024 |
| SERPINB3 | 0.00314053 | 180.6124997 |
| SH3GLB2 | 0.00389444 | 0.392616496 |
| FXYD6 | 0.044884191 | 0.406000664 |
| KLK1 | 0.004179923 | 0.345589554 |
| CD86 | 0.039690828 | 2.635583007 |
| LAMP5 | 0.025397866 | 2.669754746 |
| TMEM8A | 0.006741226 | 2.446106752 |
| WNT6 | 0.020901203 | 0.383182879 |
| SUB1 | 0.002303288 | 3.206227789 |
| C21orf62 | 0.022787663 | 0.424518073 |
| IFIT5 | 0.014959595 | 2.582026221 |
| SLC39A3 | 0.009569698 | 2.189417189 |
| ZC3H6 | 0.020167931 | 0.422301 |
| NDUFB6 | 0.004811525 | 2.165524527 |
| MYO1F | 0.017733784 | 2.199332415 |
| GSTM3 | 0.015851895 | 0.434884905 |
| CDKN3 | 0.001086144 | 3.449283257 |
| IVL | 0.038763642 | 5.785095982 |
| SLC52A3 | 0.007539643 | 4.008572646 |
| MAP3K9 | 0.003923738 | 2.373101198 |
| NDC80 | 0.002891297 | 2.686691623 |
| BCAM | 0.014382352 | 0.408447258 |
| LSM10 | 0.00778292 | 2.102379631 |
| MYBL2 | 0.0105813 | 3.979176816 |
| NOL6 | 0.00029584 | 3.357093817 |
| POLR3G | 0.003574064 | 4.211219323 |
| ZBTB16 | 0.001424199 | 0.078037266 |
| MYCBP | 0.004827367 | 2.430902829 |
| TNFRSF19 | 0.023842432 | 0.322848047 |
| CHEK1 | 0.008735651 | 2.524244552 |
| SLC51A | 0.029989731 | 2.519816444 |
| NEIL3 | 0.000695449 | 2.320497831 |
| NBPF8 | 0.008925172 | 0.448522109 |
| IFIT2 | 0.007797205 | 3.838755058 |
| FOLH1 | 0.01385732 | 3.027808617 |
| NOXA1 | 0.028871887 | 0.494963901 |
| KIAA1683 | 0.004127308 | 0.21163936 |
| GSTT2B | 0.004305897 | 0.303485922 |
| TNFSF13B | 0.035266629 | 2.676454334 |
| CAPZA3 | 0.00960603 | 0.199336527 |
| MILR1 | 0.018536345 | 3.572488454 |
| SLC39A2 | 0.016597251 | 3.023940104 |
| GM2A | 0.005671427 | 3.138232965 |
| IFI44L | 0.017928792 | 7.328285801 |
| PITPNM3 | 0.031765706 | 0.422293698 |
| MEDAG | 0.040938901 | 4.435310123 |
| KIAA0101 | 0.014008916 | 4.265184601 |
| IL12RB1 | 0.008320577 | 11.211012 |
| NWD2 | 0.011547338 | 9.564000218 |
| KCNK6 | 0.010707836 | 2.096536297 |
| PDZD7 | 0.022912279 | 0.391998973 |
| UBN2 | 0.015418919 | 0.404217696 |
| SLC27A4 | 0.020447932 | 2.316407387 |
| FUT2 | 0.000875073 | 8.030988824 |
| GAL3ST1 | 0.011600552 | 0.224822356 |
| KYNU | 0.003435937 | 15.85486251 |
| ERG | 0.004281133 | 7.145818414 |
| CEMIP | 0.004470398 | 6.524277237 |
| ELOVL7 | 0.013298089 | 3.439667304 |
| C21orf2 | 0.026327887 | 0.446047928 |
| FAM189A2 | 0.010720641 | 0.227918155 |
| JAK3 | 0.014652209 | 2.641655297 |
| CXCL13 | 0.013206063 | 55.40964534 |
| C12orf75 | 0.03771667 | 2.322603132 |
| IGFL1 | 0.011148743 | 75.64659923 |
| OIP5 | 0.004151863 | 2.830141587 |
| GTSE1 | 0.008858098 | 3.790685949 |
| SYT8 | 0.039602329 | 0.170607203 |
| PLLP | 0.032078581 | 0.456408709 |
| SORCS2 | 0.007251328 | 2.204853765 |
| IDO1 | 0.018206228 | 19.73822213 |
| AGFG2 | 0.014010283 | 0.3607785 |
| SLC39A7 | 0.004466004 | 2.376401762 |
| SLC19A1 | 0.011426299 | 2.057061362 |
| ZWINT | 0.000221667 | 2.992511444 |
| RAB38 | 0.007498084 | 2.159636638 |
| APLN | 0.014966148 | 3.708163113 |
| FOXP3 | 0.04263269 | 3.516753975 |
| ISG15 | 0.001563411 | 10.77895841 |
| SDR9C7 | 0.016589237 | 3.432021566 |
| MAPK3 | 0.002563912 | 2.067304666 |
| MAML2 | 0.046676377 | 0.470465811 |
| HIST1H2BJ | 0.001884994 | 4.338556756 |
| PPP3R1 | 0.001586099 | 0.293710147 |
| S100A7 | 0.047660911 | 28.37382209 |
| POGZ | 0.014388746 | 0.455774543 |
| ARSF | 0.007017864 | 13.88660392 |
| RASGRF1 | 0.045219991 | 2.468763863 |
| COX19 | 0.016478109 | 0.455417223 |
| FAP | 0.046842024 | 3.006931552 |
| PLEKHG5 | 0.009000979 | 0.283458717 |
| TMEM205 | 0.034076051 | 0.371511191 |
| COL21A1 | 0.027278027 | 0.423706185 |
| DCBLD1 | 0.01756441 | 2.283362038 |
| IL12RB2 | 0.002123972 | 4.188012853 |
| CEP78 | 0.038844984 | 2.463000509 |
| SEC24D | 0.007354136 | 2.278964555 |
| S100A8 | 0.021140404 | 53.54776306 |
| LRIG3 | 0.0032988 | 0.464476864 |
| CLCN3 | 0.008759582 | 2.154273392 |
| SRM | 0.002309931 | 3.054944606 |
| IKBIP | 0.032516692 | 2.24051527 |
| XPC | 0.006293721 | 0.377695433 |
| PRKAG1 | 0.001873246 | 2.165129461 |
| KLK8 | 0.013988719 | 2.189689997 |
| MRPS31 | 0.006734795 | 2.067631744 |
| GSTA5 | 0.011372196 | 0.38419854 |
| GPR18 | 0.039065217 | 4.6850693 |
| CDC42BPG | 0.010195244 | 0.432074127 |
| NOTCH4 | 0.003236161 | 15.44755263 |
| RNASE7 | 0.002664145 | 6.337174822 |
| SASH3 | 0.023600552 | 4.133686083 |
| RGL4 | 0.022496912 | 3.095396383 |
| HOXD9 | 0.002004681 | 0.445873606 |
| FOXO6 | 0.01251052 | 0.253764775 |
| TMEM70 | 0.00227527 | 2.010481852 |
| EXO1 | 0.005475898 | 7.203620037 |
| OBSCN | 0.026378349 | 0.379311866 |
| MKKS | 0.009110423 | 2.410102526 |
| ABCA3 | 0.023953243 | 0.393108092 |
| FAM157B | 0.002998326 | 2.564559396 |
| RORC | 0.003222378 | 0.270097266 |
| NRIP1 | 0.022133253 | 2.232599925 |
| C22orf29 | 0.004232005 | 0.461861798 |
| GALNT6 | 0.000601806 | 5.421718033 |
| CADM2 | 0.02199297 | 0.251083001 |
| ARHGDIB | 0.015595811 | 2.939366431 |
| NTF4 | 0.022944656 | 0.375988908 |
| ANKZF1 | 0.027077304 | 0.456877413 |
| TCEAL6 | 0.024618939 | 0.498809451 |
| ARPC4 | 0.00142202 | 2.031030616 |
| STK39 | 0.020101783 | 2.129889152 |
| SGPP2 | 0.044369116 | 2.317402122 |
| PINX1 | 0.003239079 | 2.409551369 |
| CNTN4 | 0.012527039 | 0.407186146 |
| TMEM165 | 0.001814055 | 3.693965891 |
