# Supplementary material for: Downregulation of miRNA miR-1305 and upregulation of miRNA miR-6785-5p may be associated with psoriasis
Source: Front Genet. 2022 Aug 10;13:891465. doi: 10.3389/fgene.2022.891465 (PMC9399421; doi:10.3389/fgene.2022.891465)
Supplement: Supplementary file 5 [file Table5.DOCX]

Table S5: MiRNA-mRNA networks of miR-1305.

| miRNA | mRNA |
| --- | --- |
| miRNA-1305 | IL23A |
| miRNA-1305 | CXCL11 |
| miRNA-1305 | CCL8 |
| miRNA-1305 | TMOD1 |
| miRNA-1305 | MEI1 |
| miRNA-1305 | ACE2 |
| miRNA-1305 | IFI16 |
| miRNA-1305 | RAB31 |
| miRNA-1305 | PAX9 |
| miRNA-1305 | ILF2 |
| miRNA-1305 | SLC25A5 |
| miRNA-1305 | FBXO5 |
| miRNA-1305 | PTPN22 |
| miRNA-1305 | KIF23 |
| miRNA-1305 | SLAMF7 |
| miRNA-1305 | GDA |
| miRNA-1305 | PNO1 |
| miRNA-1305 | RMI2 |
| miRNA-1305 | CXCR2 |
| miRNA-1305 | PLOD2 |
| miRNA-1305 | GPRIN3 |
| miRNA-1305 | STRN3 |
| miRNA-1305 | EIF1AX |
| miRNA-1305 | EHF |
| miRNA-1305 | KIF4A |
| miRNA-1305 | MAN2A1 |
| miRNA-1305 | TEX30 |
| miRNA-1305 | ZWILCH |
| miRNA-1305 | TFEC |
| miRNA-1305 | NRIP1 |
| miRNA-1305 | RGS20 |
| miRNA-1305 | SELE |
| miRNA-1305 | ARPC1B |
| miRNA-1305 | MXD1 |
| miRNA-1305 | POLR3G |
| miRNA-1305 | AMMECR1 |
| miRNA-1305 | ITGB6 |
| miRNA-1305 | PRICKLE2 |
| miRNA-1305 | TRPM6 |
| miRNA-1305 | HIF1A |
| miRNA-1305 | KBTBD8 |
| miRNA-1305 | SUB1 |
| miRNA-1305 | ADAMTS5 |
| miRNA-1305 | RND3 |
| miRNA-1305 | SLC24A4 |
| miRNA-1305 | MID1 |
| miRNA-1305 | PTP4A1 |
| miRNA-1305 | PGM2 |
| miRNA-1305 | PRSS53 |
| miRNA-1305 | SLC26A4 |
| miRNA-1305 | TMPRSS11D |
| miRNA-1305 | THBD |
| miRNA-1305 | MYCBP |
| miRNA-1305 | HEATR5A |
| miRNA-1305 | ENTPD7 |
| miRNA-1305 | GALNT13 |
| miRNA-1305 | SLC25A13 |
| miRNA-1305 | UCK2 |
| miRNA-1305 | STK39 |
| miRNA-1305 | SLC7A11 |
| miRNA-1305 | GGH |
| miRNA-1305 | MTHFD2 |
| miRNA-1305 | RALA |
| miRNA-1305 | HTR7 |
| miRNA-1305 | ZFAND6 |
| miRNA-1305 | DUS2 |
| miRNA-1305 | NRBF2 |
| miRNA-1305 | EAF1 |
| miRNA-1305 | CCNYL1 |
| miRNA-1305 | ARL5B |
| miRNA-1305 | CLCN3 |
| miRNA-1305 | WDR76 |
| miRNA-1305 | CCNB1 |
| miRNA-1305 | C12orf29 |
| miRNA-1305 | TSFM |
| miRNA-1305 | ATP1B1 |
| miRNA-1305 | LILRB2 |
