# Supplementary material for: Downregulation of miRNA miR-1305 and upregulation of miRNA miR-6785-5p may be associated with psoriasis
Source: Front Genet. 2022 Aug 10;13:891465. doi: 10.3389/fgene.2022.891465 (PMC9399421; doi:10.3389/fgene.2022.891465)
Supplement: Supplementary file 6 [file Table3.DOCX]

Table S3: upregulated mRNAs might be regulated by miR-1305

| mRNA |
| --- |
| IL23A |
| CXCL11 |
| CCL8 |
| TMOD1 |
| MEI1 |
| ACE2 |
| IFI16 |
| RAB31 |
| PAX9 |
| ILF2 |
| SLC25A5 |
| FBXO5 |
| PTPN22 |
| KIF23 |
| SLAMF7 |
| GDA |
| PNO1 |
| RMI2 |
| CXCR2 |
| PLOD2 |
| GPRIN3 |
| STRN3 |
| EIF1AX |
| EHF |
| KIF4A |
| MAN2A1 |
| TEX30 |
| ZWILCH |
| TFEC |
| NRIP1 |
| RGS20 |
| SELE |
| ARPC1B |
| MXD1 |
| POLR3G |
| AMMECR1 |
| ITGB6 |
| PRICKLE2 |
| TRPM6 |
| HIF1A |
| KBTBD8 |
| SUB1 |
| ADAMTS5 |
| RND3 |
| SLC24A4 |
| MID1 |
| PTP4A1 |
| PGM2 |
| PRSS53 |
| SLC26A4 |
| TMPRSS11D |
| THBD |
| MYCBP |
| HEATR5A |
| ENTPD7 |
| GALNT13 |
| SLC25A13 |
| UCK2 |
| STK39 |
| SLC7A11 |
| GGH |
| MTHFD2 |
| RALA |
| HTR7 |
| ZFAND6 |
| DUS2 |
| NRBF2 |
| EAF1 |
| CCNYL1 |
| ARL5B |
| CLCN3 |
| WDR76 |
| CCNB1 |
| C12orf29 |
| TSFM |
| ATP1B1 |
| LILRB2 |
