# Supplementary material for: Downregulation of miRNA miR-1305 and upregulation of miRNA miR-6785-5p may be associated with psoriasis
Source: Front Genet. 2022 Aug 10;13:891465. doi: 10.3389/fgene.2022.891465 (PMC9399421; doi:10.3389/fgene.2022.891465)
Supplement: Supplementary file 7 [file Table4.DOCX]

Table S4: Downregulated mRNAs might be regulated by miR-6785-5p.

| mRNA |
| --- |
| SYNGR1 |
| SOX12 |
| GPC1 |
| TOM1L2 |
| HIF3A |
| FBXL16 |
| RAPGEFL1 |
| AHDC1 |
| NR1D1 |
| CNTFR |
| PHYHIP |
| CYP2W1 |
| RAD51B |
| PPM1J |
| KY |
| XKR6 |
| COX6B2 |
| P2RX6 |
| ESPN |
| SH3GLB2 |
| KCNJ12 |
| WNT3A |
| RTN4RL1 |
| IQSEC2 |
| CSAD |
| ZC3H7B |
| ARPC4-TTLL3 |
| APCDD1 |
| FOXO6 |
| CACNA2D2 |
| ZNF703 |
| APBA1 |
| VWA2 |
| TMEM63C |
| GAL3ST1 |
| RABL2A |
| LFNG |
| PITPNM3 |
| ZNF490 |
| CHRM1 |
| BSDC1 |
| CHAD |
| SLC47A1 |
| MLXIP |
| MCF2L |
| B3GAT3 |
| PLLP |
| EXPH5 |
| MAP6 |
| SHC2 |
| BCAM |
| NRF1 |
| ZNF793 |
| GTF3C1 |
| DNAJC30 |
| RAB40C |
| CGNL1 |
| SGSM1 |
| ZNF320 |
| PER1 |
| EEF2K |
| NEUROD2 |
| GPR17 |
| RBM20 |
| CACNA2D1 |
| ZNF528 |
| ZNF471 |
| PTPRU |
| PLEKHH1 |
| CACNG8 |
| CACNA1H |
| WSCD1 |
| ELFN2 |
| OBSCN |
| IL17RD |
| ZC3H6 |
| WNT4 |
| PAIP2B |
| KIFC2 |
| ZNF43 |
| LAMC3 |
| HYI |
| ELAVL1 |
