# Supplementary material for: Downregulation of miRNA miR-1305 and upregulation of miRNA miR-6785-5p may be associated with psoriasis
Source: Front Genet. 2022 Aug 10;13:891465. doi: 10.3389/fgene.2022.891465 (PMC9399421; doi:10.3389/fgene.2022.891465)
Supplement: Supplementary file 8 [file Table6.DOCX]

Table S6: MiRNA-mRNA networks of miR-6785-5p.

| miRNA | mRNA |
| --- | --- |
| miR-6785-5p | SYNGR1 |
| miR-6785-5p | SOX12 |
| miR-6785-5p | GPC1 |
| miR-6785-5p | TOM1L2 |
| miR-6785-5p | HIF3A |
| miR-6785-5p | FBXL16 |
| miR-6785-5p | RAPGEFL1 |
| miR-6785-5p | AHDC1 |
| miR-6785-5p | NR1D1 |
| miR-6785-5p | CNTFR |
| miR-6785-5p | PHYHIP |
| miR-6785-5p | CYP2W1 |
| miR-6785-5p | RAD51B |
| miR-6785-5p | PPM1J |
| miR-6785-5p | KY |
| miR-6785-5p | XKR6 |
| miR-6785-5p | COX6B2 |
| miR-6785-5p | P2RX6 |
| miR-6785-5p | ESPN |
| miR-6785-5p | SH3GLB2 |
| miR-6785-5p | KCNJ12 |
| miR-6785-5p | WNT3A |
| miR-6785-5p | RTN4RL1 |
| miR-6785-5p | IQSEC2 |
| miR-6785-5p | CSAD |
| miR-6785-5p | ZC3H7B |
| miR-6785-5p | ARPC4-TTLL3 |
| miR-6785-5p | APCDD1 |
| miR-6785-5p | FOXO6 |
| miR-6785-5p | CACNA2D2 |
| miR-6785-5p | ZNF703 |
| miR-6785-5p | APBA1 |
| miR-6785-5p | VWA2 |
| miR-6785-5p | TMEM63C |
| miR-6785-5p | GAL3ST1 |
| miR-6785-5p | RABL2A |
| miR-6785-5p | LFNG |
| miR-6785-5p | PITPNM3 |
| miR-6785-5p | ZNF490 |
| miR-6785-5p | CHRM1 |
| miR-6785-5p | BSDC1 |
| miR-6785-5p | CHAD |
| miR-6785-5p | SLC47A1 |
| miR-6785-5p | MLXIP |
| miR-6785-5p | MCF2L |
| miR-6785-5p | B3GAT3 |
| miR-6785-5p | PLLP |
| miR-6785-5p | EXPH5 |
| miR-6785-5p | MAP6 |
| miR-6785-5p | SHC2 |
| miR-6785-5p | BCAM |
| miR-6785-5p | NRF1 |
| miR-6785-5p | ZNF793 |
| miR-6785-5p | GTF3C1 |
| miR-6785-5p | DNAJC30 |
| miR-6785-5p | RAB40C |
| miR-6785-5p | CGNL1 |
| miR-6785-5p | SGSM1 |
| miR-6785-5p | ZNF320 |
| miR-6785-5p | PER1 |
| miR-6785-5p | EEF2K |
| miR-6785-5p | NEUROD2 |
| miR-6785-5p | GPR17 |
| miR-6785-5p | RBM20 |
| miR-6785-5p | CACNA2D1 |
| miR-6785-5p | ZNF528 |
| miR-6785-5p | ZNF471 |
| miR-6785-5p | PTPRU |
| miR-6785-5p | PLEKHH1 |
| miR-6785-5p | CACNG8 |
| miR-6785-5p | CACNA1H |
| miR-6785-5p | WSCD1 |
| miR-6785-5p | ELFN2 |
| miR-6785-5p | OBSCN |
| miR-6785-5p | IL17RD |
| miR-6785-5p | ZC3H6 |
| miR-6785-5p | WNT4 |
| miR-6785-5p | PAIP2B |
| miR-6785-5p | KIFC2 |
| miR-6785-5p | ZNF43 |
| miR-6785-5p | LAMC3 |
| miR-6785-5p | HYI |
| miR-6785-5p | ELAVL1 |
